# Early CYNC-1 Administration Attenuates Radiation-Induced Lung Inflammation and Subsequent Fibrotic Remodeling

**DOI:** 10.3390/pharmaceutics18101321

**Published:** 2026-10-18

**Authors:** Youn Ji Hur, Jae-Ho Shin, Sang Yeon Kim, Ronglan Cui, Mohamed El-Agamy Farh, Sun Ho Min, Woo Hyun Jeong, Sunjoo Park, Younghwa Na, Jaeho Cho

**Affiliations:** 1 Department of Radiation Oncology, Yonsei University College of Medicine, Seoul 03722, Republic of Korea; hyj@yuhs.ac (Y.J.H.); sysykim@honam.ac.kr (S.Y.K.); choiyr322@yuhs.ac (R.C.); shmin610@yuhs.ac (S.H.M.); 2 College of Pharmacy, CHA University, Pocheon-Si 11160, Gyeonggi-Do, Republic of Korea; wogh975@naver.com (J.-H.S.); dngus3335@naver.com (W.H.J.); yna7315@cha.ac.kr (Y.N.); 3 Department of Food and Nutrition, College of Health Science, Honam University, Gwangju 62399, Republic of Korea; 4 AI-Bio Convergence Research Institute, Soongsil University, Seoul 06978, Republic of Korea; farhmohamed1989@gmail.com

**Keywords:** radiation-induced lung injury, synthetic lipoxin A4 mimetic, chemical stability, pharmacological activity, inflammation, pulmonary fibrosis

## Abstract

**Background/Objectives:** Radiation-induced lung injury (RILI) involves inflammatory responses followed by fibrotic remodeling. We evaluated CYNC-1, a synthetic compound designed as a lipoxin A4 (LXA4) mimetic, for chemical stability, cellular activity, and attenuation of lung injury after irradiation. **Methods:** HeLa-derived L132 epithelial-like cells and THP1-Lucia NF-κB reporter monocytes were used for cell-based assays. Male C57BL/6 mice received a single 75-Gy focal lung irradiation. CYNC-1 administration began immediately after irradiation and continued every other day for 2 or 6 weeks. Lung injury was assessed using bronchoalveolar lavage fluid cellularity, micro-computed tomography, histopathology, and lung mechanics. Prednisolone and nintedanib served as reference treatments in the 2- and 6-week cohorts, respectively. **Results:** CYNC-1 maintained high HPLC area purity during light-protected ethanol storage for 48 h at −78, −20, and 4 °C. It increased survival of irradiated L132 cells and reduced LPS-induced NF-κB reporter activity. In L132 cells, treatment was associated with reduced NF-κB/MAPK and Smad2 phosphorylation and lower profibrotic marker expression. In mice, CYNC-1 reduced inflammatory cell accumulation and collagen deposition and improved lung structure and mechanics. **Conclusions:** Early and continued CYNC-1 administration attenuated lung inflammation and subsequent fibrotic remodeling in this focal irradiation model. The signaling findings are associative, and FPR2 dependence and efficacy against established fibrosis remain to be established. Validation in primary human lung cells, clinically relevant fractionated irradiation models, animals of both sexes, and tumor models is needed to determine suitability as a normal-tissue protectant, including preservation of antitumor efficacy.

## 1. Introduction

Radiotherapy (RT) is an essential component of curative and palliative treatment for thoracic malignancies, particularly lung cancer. Despite advances in treatment planning and delivery that have improved dose conformity and reduced unnecessary irradiation of normal tissues, radiation-induced lung injury (RILI) remains a major dose-limiting toxicity of thoracic RT. RILI represents a dynamic continuum that generally manifests as an early inflammatory phase, clinically recognized as radiation pneumonitis, followed by a delayed fibrotic phase characterized by irreversible tissue remodeling and progressive impairment of pulmonary function [1,2,3]. Depending on the patient population, treatment regimen, and diagnostic criteria, clinically apparent RILI occurs in approximately 10–30% of patients receiving thoracic RT [1,2].

Current treatment options remain limited. Systemic corticosteroids are the mainstay of treatment for symptomatic radiation pneumonitis; however, they primarily suppress the inflammatory response, frequently require prolonged tapering, and do not reliably prevent subsequent fibrotic remodeling [1,4]. Antifibrotic agents such as nintedanib have demonstrated activity in preclinical models and encouraging clinical signals, but their efficacy in RILI has not been definitively established [5,6,7]. Therefore, therapeutic strategies capable of modulating both the inflammatory and fibrotic phases of RILI are needed.

The biological response to ionizing radiation begins within minutes through the generation of reactive oxygen species and reactive nitrogen species, resulting in DNA damage, mitochondrial dysfunction, and injury or death of alveolar epithelial and vascular endothelial cells [1,3,8]. Damaged cells release damage-associated molecular patterns and inflammatory mediators that activate resident macrophages and promote the recruitment of neutrophils, lymphocytes, and other immune cells [3,8,9]. These events induce the production of cytokines and chemokines, including tumor necrosis factor-α, interleukin (IL)-1, IL-6, CXCL8, and transforming growth factor-β (TGF-β), thereby establishing a self-amplifying inflammatory microenvironment [8,9,10,11].

Nuclear factor-κB (NF-κB) is a central transcriptional regulator of this radiation-induced inflammatory response. NF-κB activation increases the expression of inflammatory cytokines, chemokines, and adhesion molecules and promotes the recruitment and activation of immune cells [12,13]. Mitogen-activated protein kinase (MAPK) pathways, including extracellular signal-regulated kinase, c-Jun N-terminal kinase, and p38 MAPK, interact with NF-κB signaling and further enhance oxidative stress and inflammatory gene expression [12,13,14,15]. Persistent activation of these pathways contributes to failure of inflammatory resolution and progression toward chronic lung injury.

Unresolved inflammation subsequently promotes fibroblast activation, myofibroblast differentiation, and excessive deposition of extracellular matrix proteins [3,16,17]. TGF-β1 is a principal mediator of this transition. Following receptor activation, phosphorylation of Smad2 and Smad3 induces the transcription of profibrotic genes, including those encoding α-smooth muscle actin, fibronectin, and type I collagen [16,17,18,19]. Radiation-induced epithelial injury may also promote partial acquisition of mesenchymal characteristics by alveolar epithelial cells, although the quantitative contribution of epithelial-to-mesenchymal transition to the fibroblast population in vivo remains debated [18,19,20,21]. Nevertheless, activation of TGF-β/Smad signaling and loss of epithelial homeostasis are consistently associated with radiation-induced pulmonary fibrosis. These observations suggest that effective mitigation of RILI may require simultaneous control of inflammatory signaling and fibrotic remodeling.

Lipoxin A4 (LXA4) is an endogenous specialized pro-resolving lipid mediator derived from arachidonic acid [22,23,24,25]. In contrast to conventional anti-inflammatory agents that broadly suppress immune activity, LXA4 promotes the active resolution of inflammation by limiting neutrophil recruitment, modulating macrophage responses, facilitating clearance of apoptotic cells, and restoring tissue homeostasis [22,23,24,25,26]. Many of these effects are mediated through formyl peptide receptor 2, also known as ALX/FPR2, although additional receptor-dependent and receptor-independent mechanisms have been described [25,27,28].

LXA4 also exerts antioxidant and antifibrotic effects. In experimental models, LXA4 reduces reactive oxygen species generation and inflammatory cytokine production and modulates NF-κB activity [24,29,30]. Of particular relevance to RILI, LXA4–FPR2 signaling has been shown to attenuate radiation-induced pulmonary inflammation and fibrosis through suppression of NF-κB activation and crosstalk with the TGF-β/Smad pathway [31]. However, the clinical application of native LXA4 is limited by rapid enzymatic inactivation, a short biological half-life, and poor chemical stability [25,32,33].

Several generations of synthetic lipoxin mimetics have therefore been developed to improve metabolic stability and retain pro-resolving biological activity [32,33,34,35,36,37]. Early approaches incorporated modifications such as methyl, fluorophenoxy, aromatic, or benzo substitutions, whereas more recent compounds have used structurally distinct scaffolds to improve pharmacological and synthetic properties. Although these analogues have demonstrated anti-inflammatory and antifibrotic activity in multiple experimental settings, their translational development has been constrained by variable potency, receptor activity, metabolic properties, and synthetic complexity [32,33,34,35]. These considerations motivated evaluation of a structurally modified compound designed as an LXA4 mimetic for attenuation of early inflammatory injury and subsequent fibrotic remodeling after irradiation. The present study examined HPLC purity profiles of CYNC-1 and native LXA4 during storage and evaluated cytoprotective and anti-inflammatory activity in the assays used.

We therefore evaluated CYNC-1 using cell-based assays and a murine focal high-dose lung irradiation model. In mice, treatment was initiated immediately after irradiation to assess attenuation of early inflammatory injury and subsequent fibrotic remodeling. We also examined whether cellular treatment effects were accompanied by changes in NF-κB/MAPK and TGF-β/Smad2-associated signaling. Prednisolone and nintedanib were included as reference treatments in the 2- and 6-week cohorts, respectively.

## 2. Materials and Methods

### 2.1. CYNC-1 Synthesis, NMR Characterization, and HPLC Analysis

Boronic acid ester intermediate 1 was synthesized according to the procedure reported by de Gaetano et al. [33]. CYNC-1 was subsequently synthesized through a palladium-catalyzed Suzuki–Miyaura cross-coupling reaction between bromobenzene and intermediate 1. Briefly, bromobenzene (50 mg, 0.32 mmol, TCI Chemicals, Tokyo, Japan), boronic acid ester compound 1 (169 mg, 0.48 mmol), potassium carbonate (9 mg, 0.06 mmol, Daejung Chemicals & Metals Co., Ltd., Siheung, Republic of Korea), and tetrakis(triphenylphosphine)palladium(0) (5 mg, 10 wt%, Combi-Blocks, San Diego, CA, USA) were reacted in a mixture of 1,4-dioxane (2 mL) and distilled water (0.2 mL) under an argon atmosphere using an Initiator+ microwave synthesizer (Biotage Sweden AB, Uppsala, Sweden) at 100 °C for 1 h. After cooling to room temperature, the reaction mixture was extracted with ethyl acetate and water. The organic phase was dried over anhydrous magnesium sulfate, concentrated under reduced pressure, and purified by silica gel column chromatography (ethyl acetate/n-hexane = 1:6, Merck Kieselgel 60 (0.040–0.063 mm), Merck KGaA, Darmstadt, Germany) to afford CYNC-1 as a semisolid product (52 mg, 53.4% yield).

CYNC-1 showed an Rf value of 0.38 on thin-layer chromatography (ethyl acetate/n-hexane = 1:6, Merck Kieselgel 60 F254 plates (0.25 mm), Merck KGaA, Darmstadt, Germany), and HPLC analysis showed a retention time of 5.13 min and a purity of 99.7%. Analytical HPLC was performed using an LC-2050C 3D system with an SPD-M20A diode-array detector (Shimadzu Corporation, Kyoto, Japan) and a SunFire C18 column (4.6 × 150 mm, 5 µm; Waters Corporation, Milford, MA, USA). CYNC-1 was eluted isocratically with water/acetonitrile (30:70, *v*/*v*) at 1.0 mL/min for 15 min. These measurements describe chromatographic behavior and purity.

Spectroscopic characterization of CYNC-1 was performed using a Varian AS 400 NMR spectrometer (Varian, Inc., Palo Alto, CA, USA) at Ewha Womans University. The ^1^H and ^13^C spectra were acquired in DMSO at 25 °C; the acquisition records specify frequencies of 400.035 and 100.600 MHz, respectively. Chemical shifts are expressed in parts per million relative to tetramethylsilane as the internal standard. The spectra and acquisition parameters are provided in Supplementary Figures S1 and S2 and Table S1, respectively.

Native lipoxin A4 (nLXA4; catalog no. 90410) was purchased from Cayman Chemical (Ann Arbor, MI, USA). For the ethanol-storage assessment, native LXA4 (nLXA4) and CYNC-1 were prepared separately in ethanol and stored in light-protective containers without buffer. nLXA4 was tested at 142 µM (0.005%, *w*/*v*; 50 µg/mL) and CYNC-1 at 164 µM (0.005%, *w*/*v*; 50 µg/mL) at −78, −20, and 4 °C. At each temperature, one vial was sampled consecutively at 3, 6, and 9 h and a second vial at 24 and 48 h. Samples were brought to room temperature after retrieval and promptly analyzed by a single manual 10 µL injection at each sampling time. The baseline and time-course values are reported in Supplementary Table S2B.

Storage samples were analyzed using the same HPLC system and column, with isocratic water/acetonitrile at 40:60 (*v*/*v*) for nLXA4 and 30:70 (*v*/*v*) for CYNC-1, a flow rate of 1.0 mL/min, and a 15 min run. No buffer was used in the mobile phase. Although nLXA4 had an absorption maximum near 300 nm, impurities were observed at 254 nm; therefore, both compounds were evaluated at 254 nm. Purity was expressed as chromatographic area percentage (Area %), calculated as 100 × the analyte peak area divided by the sum of integrated peak areas in the 254 nm chromatogram. Area % was not normalized to the initial amount of compound. Repeated sampling from the same vial did not provide independent replicate samples. This unbuffered ethanol-storage assessment did not assess controlled-pH stability or photostability. The analytical conditions and the storage protocol are detailed in Supplementary Table S2.

### 2.2. Cell Culture

L132 cells, a HeLa-derived epithelial-like cell line, were obtained from the American Type Culture Collection (ATCC, Manassas, VA, USA) and were used for the cell-based radiation-response assays in this study. Cells were maintained in Dulbecco’s modified Eagle’s medium (DMEM; Gibco, Thermo Fisher Scientific, Waltham, MA, USA) supplemented with 10% fetal bovine serum (FBS; HyClone, Cytiva, Logan, UT, USA), 100 U/mL penicillin and 100 μg/mL streptomycin (catalog no. LS202-02; WELGENE, Gyeongsan, Republic of Korea). Cells were cultured at 37 °C in a humidified incubator containing 5% CO_2_ and were routinely passaged every 2–3 days. Only exponentially growing cells were used for all experiments. For in vitro experiments, nLXA4 and CYNC-1 were dissolved in 100% ethanol to prepare stock solutions and subsequently diluted to the indicated working concentrations in culture medium. An equivalent volume of ethanol was used for vehicle controls.

### 2.3. Cell Viability Assay

Cell metabolic activity was evaluated using the WST-1 Cell Proliferation Reagent (Dojindo Laboratories, Kumamoto, Japan) according to the manufacturer’s instructions. L132 cells were seeded in 96-well plates at a density of 1 × 10^4^ cells per well and allowed to attach overnight. To determine radiation-induced cytotoxicity, cells were exposed to single radiation doses of 0, 2, 4, 6, 8, or 10 Gy using an X-RAD320 irradiator (Precision X-Ray Inc., North Branford, CT, USA). Cell viability was measured at 24, 48, and 72 h after irradiation. To evaluate the radioprotective effects of CYNC-1, cells were pretreated with CYNC-1 or native lipoxin A_4_ (nLXA_4_) at concentrations ranging from 1 to 1000 nM for 2 h before exposure to 8 Gy irradiation. Cell viability was assessed 24 h after irradiation by adding WST-1 reagent according to the manufacturer’s protocol, followed by incubation for 2 h at 37 °C. Absorbance was measured at 450 nm using a SUNRISE microplate reader (Tecan, Männedorf, Switzerland). Cell viability was expressed as the percentage of the corresponding non-irradiated control. All experiments were independently repeated at least three times with triplicate technical replicates.

### 2.4. Colony Formation Assay

L132 cells were seeded into 6-well plates at densities optimized for each radiation dose (6 × 10^2^, 1.8 × 10^3^, and 4.8 × 10^3^ cells per well) to ensure adequate colony formation. After overnight attachment, cells were pretreated with native lipoxin A_4_ (nLXA_4_) or CYNC-1 for 2 h and exposed to single doses of 0, 2, 4, 6, 8, or 10 Gy using an X-RAD320 irradiator (Precision X-Ray Inc., North Branford, CT, USA). Cells were subsequently cultured in complete DMEM containing the corresponding treatments for approximately 14 days to allow colony formation. At the end of the incubation period, colonies were fixed with methanol and stained with 0.5% crystal violet (catalog no. C0775; Sigma-Aldrich, St. Louis, MO, USA). Colonies containing ≥50 cells were considered clonogenic survivors and were automatically quantified using an ImmunoSpot^®^ Imaging Analyzer (Cellular Technology Ltd., Cleveland, OH, USA). Plating efficiency and surviving fraction were calculated using standard radiobiological methods, and all experiments were independently repeated at least three times.

### 2.5. NF-κB Reporter Gene Assay

NF-κB transcriptional activity was evaluated using THP1-Lucia™ NF-κB reporter monocytes (InvivoGen, Toulouse, France), which stably express a secreted luciferase reporter under the control of an NF-κB-inducible promoter. Cells were maintained in RPMI-1640 medium (Gibco, Thermo Fisher Scientific, Waltham, MA, USA) supplemented with 10% fetal bovine serum, 2 mM L-glutamine (catalog no. 59202C; Sigma-Aldrich, St. Louis, MO, USA), 25 mM HEPES, 100 U/mL penicillin, 100 μg/mL streptomycin, 100 μg/mL Normocin™ (catalog no. ant-nr-2, InvivoGen, Toulouse, France), and 100 μg/mL Zeocin™ (catalog no. ant-zn-1, InvivoGen, Toulouse, France) under standard culture conditions (37 °C, 5% CO_2_). For the reporter assay, cells were seeded at 1 × 10^5^ cells per well in 96-well plates containing RPMI-1640 supplemented with 0.1% fetal bovine serum to minimize basal NF-κB activity. Cells were pretreated with vehicle (0.1% ethanol (*v*/*v*)), nLXA_4_, or CYNC-1 (0.001–1000 nM) for 2 h before stimulation with lipopolysaccharide (LPS; Sigma-Aldrich, St. Louis, MO, USA; 1 μg/mL) for an additional 24 h. Cell-free culture supernatants were collected, and secreted luciferase activity was quantified using Quanti-Luc™ reagent (InvivoGen, Toulouse, France) according to the manufacturer’s instructions. Luminescence was measured using a Centro XS^3^ LB960 microplate luminometer (Berthold Technologies, Bad Wildbad, Germany). NF-κB activity was expressed as relative luminescence units normalized to the LPS-treated control group. Each experiment was independently repeated at least three times.

### 2.6. Animal Experiments and Drug Administration

All animal procedures were approved by the Institutional Animal Care and Use Committee of Yonsei University Health System (Approval Code: YUHS-IACUC-2022-0186; Approval Date: 19 August 2022), and were conducted in accordance with institutional guidelines and the ARRIVE 2.0 recommendations for reporting animal research. Male C57BL/6 mice (7 weeks of age, 20–25 g) were obtained from Orient Bio (Seongnam, Republic of Korea) and housed under specific pathogen-free conditions in a temperature- and humidity-controlled facility with a 12 h light/dark cycle and free access to standard chow and water. Following a one-week acclimatization period, mice were randomly assigned to the experimental groups. A focal radiation-induced lung injury (RILI) model was established as previously described using an X-RAD320 irradiator (Precision X-Ray Inc., North Branford, CT, USA) [38,39]. Briefly, mice received a single 75-Gy dose specifically delivered to the left lung through a 5 mm circular collimator, while the remainder of the body was shielded with lead to minimize radiation exposure to non-target tissues. This focal irradiation model reproducibly induces a biphasic pattern of lung injury characterized by an early inflammatory phase followed by progressive pulmonary fibrosis.

For each experimental time point, independent cohorts of mice (*n* = 5 per group) were used. Separate groups of animals were sacrificed at 2 weeks to evaluate the inflammatory phase and at 6 weeks to evaluate the fibrotic phase following irradiation. For in vivo experiments, CYNC-1 was dissolved in 100% ethanol as the stock solvent. The Control and IR groups received an equivalent volume of the corresponding vehicle. The experimental groups consisted of the following: (1) Control: non-irradiated mice receiving vehicle treatment; (2) IR: irradiated mice receiving vehicle treatment; (3) IR+CYNC-1: irradiated mice treated with CYNC-1 (0.5 or 2.5 mg/kg, intraperitoneally, every other day); (4) IR+Prednisolone: irradiated mice treated with prednisolone (Sigma-Aldrich, St. Louis, MO, USA; 2 mg/kg, orally, every other day for 14 days); (5) IR+Nintedanib: irradiated mice treated with nintedanib (MedChemExpress, Monmouth Junction, NJ, USA; 60 mg/kg, orally, every other day for 6 weeks). Drug treatment was initiated immediately after irradiation (Day 0). CYNC-1 was administered every other day for either 2 weeks or 6 weeks. The 6-week cohort evaluated the effects of CYNC-1 initiated immediately after irradiation and continued throughout follow-up on subsequent fibrotic remodeling. An independent preliminary dose-selection experiment was conducted before the main experiments (Supplementary Figure S3 and Table S4A–C).

At the designated experimental endpoints, mice underwent micro-computed tomography (micro-CT) imaging and pulmonary function assessment before euthanasia. Irradiated left lungs were subsequently harvested for histological, immunohistochemical, and molecular analyses. For the main in vivo experiments, no animals or tissue samples were excluded from the final analyses.

### 2.7. Micro-Computed Tomography (CT) Analysis

Structural changes in irradiated lungs were evaluated at the 2- and 6-week endpoints in independent cohorts using a high-resolution micro-computed tomography (micro-CT) system (Quantum GX2, PerkinElmer, Shelton, CT, USA). Mice were scanned under inhalational anesthesia using standardized acquisition parameters (50 kVp, 180 μA, estimated radiation dose of 150 mGy), and volumetric datasets were reconstructed into three-dimensional images using 3D Slicer (version 5.6.2; https://www.slicer.org/). Radiation-associated lung injury was quantitatively assessed using Analyze 14.0 software (AnalyzeDirect, Overland Park, KS, USA). Regions of interest corresponding to the irradiated left lung were analyzed to determine changes in lung structure and tissue density. Image analyses were performed using identical analysis parameters for all animals while blinded to treatment allocation.

### 2.8. Functional Assessment of the Lung

Pulmonary function was evaluated using the FlexiVent™ FX system (SCIREQ, Montreal, QC, Canada) based on the forced oscillation technique, according to the manufacturer’s recommendations. Mice were anesthetized, tracheostomized, mechanically ventilated, and subjected to standardized respiratory perturbations to assess both airway and parenchymal mechanics. Respiratory system mechanics were analyzed using both the single-compartment and constant-phase models. The single-compartment model was used to determine respiratory system resistance (Rrs), compliance (Crs), and elastance (Ers). The constant-phase model was applied to calculate Newtonian resistance (Rn), tissue damping (G), and tissue elastance (H), allowing discrimination between central airway resistance and peripheral lung tissue mechanics.

### 2.9. Histological, Immunohistochemical and Morphometric Analyses

Following euthanasia, irradiated left lungs were harvested, fixed in 4% paraformaldehyde, dehydrated through graded ethanol, embedded in paraffin, and sectioned at a thickness of 4 μm. Tissue sections were stained with hematoxylin and eosin (H&E) to evaluate inflammatory changes and with Masson’s trichrome (MT; Abcam, Cambridge, UK) to assess collagen deposition and pulmonary fibrosis. Histopathological alterations, including inflammatory cell infiltration, bronchiolar epithelial thickening, vascular wall thickening, and collagen accumulation, were independently evaluated by experienced pathologists who were blinded to the experimental groups. Morphometric quantification was performed using ImageJ software (version 1.54u; National Institutes of Health, Bethesda, MD, USA), and identical analysis parameters were applied to all specimens.

For immunohistochemical analysis, deparaffinized tissue sections underwent antigen retrieval followed by overnight incubation at 4 °C with primary antibodies against phospho-NF-κB p65 (Ser536; catalog no. 3033), phospho-p38 MAPK (Thr180/Tyr182; catalog no. 4511), and α-smooth muscle actin (α-SMA; clone D4K9N; catalog no. 19245S), all from Cell Signaling Technology (Danvers, MA, USA), and against fibronectin (catalog no. ab2413; Abcam, Cambridge, UK). Immunoreactivity was detected using the Dako REAL EnVision Detection System, Peroxidase/DAB+, Rabbit/Mouse (catalog no. K5007; Dako, Agilent Technologies, Glostrup, Denmark), with the supplied DAB+ chromogen. Stained sections were digitally scanned, and representative images were acquired under identical microscope settings for all experimental groups.

### 2.10. RNA-seq Data Analysis

To characterize the temporal molecular landscape of radiation-induced lung injury, we reanalyzed the previously published RNA-seq dataset reported by Farh et al. [40] and deposited in the Gene Expression Omnibus (GEO; accession GSE205025). The analysis used lung samples from male C57BL/6 mice exposed to focal 75-Gy irradiation and time-matched non-irradiated controls at 2 and 6 weeks, with three independent mice per group (12 samples in total). Each sample represented RNA from one mouse; no RNA pooling was performed. The included samples were GSM6204267–GSM6204269 (non-irradiated, 2 weeks; sample labels 3126–3128), GSM6204273–GSM6204275 (75 Gy, 2 weeks; 3132–3134), GSM6204276–GSM6204278 (non-irradiated, 6 weeks; 3135–3137), and GSM6204281–GSM6204283 (75 Gy, 6 weeks; 3141–3143). The 65-Gy groups in the source dataset were not included. This secondary analysis provides radiation-model context and does not evaluate the effects of CYNC-1 treatment.

For the descriptive heatmap, read counts for 23 selected genes related to inflammatory signaling, extracellular matrix remodeling, and fibrogenesis were extracted from the source dataset. Heatmaps were generated using the heatmap.2 function in the gplots package (version 3.1.3.1) in R (version 4.4.1; R Foundation for Statistical Computing, Vienna, Austria). For visualization, counts were standardized within each gene across the 12 samples by subtracting the row mean and dividing by the row standard deviation (row z-scores; scale = “row”). No additional library-size normalization or log transformation was applied in the heatmap workflow. Genes were hierarchically clustered using a distance of 1 minus the Pearson correlation coefficient and average linkage; columns retained the predefined sample order. The heatmap is descriptive and does not represent log_2_ fold changes, a differential-expression test, or a pathway-enrichment analysis.

### 2.11. Quantitative Real-Time Polymerase Chain Reaction (RT-qPCR)

Total RNA was extracted from L132 cells using TRIzol™ reagent (Invitrogen, Carlsbad, CA, USA) according to the manufacturer’s instructions. Cells were pretreated with nLXA_4_ or CYNC-1 (1 or 10 nM) for 2 h before exposure to 6 Gy irradiation and were harvested 24 h after irradiation. Complementary DNA (cDNA) was synthesized from total RNA using the High-Capacity cDNA Reverse Transcription Kit (Applied Biosystems, Foster City, CA, USA). Quantitative real-time PCR was performed using iQ™ SYBR Green Supermix (Bio-Rad Laboratories, Hercules, CA, USA) on a CFX real-time PCR system (Bio-Rad Laboratories). Each 20-μL reaction contained SYBR Green master mix, gene-specific primers (Supplementary Table S3), nuclease-free water, and cDNA template. Relative mRNA expression levels were normalized to GAPDH and calculated using the 2^−ΔΔCt^ method.

The expression of representative inflammatory cytokines (TNF, IL1B, IL6, and CXCL8), the profibrotic cytokine TGFB1, and the anti-inflammatory cytokine IL10 was analyzed. All reactions were performed in triplicate from at least three independent biological experiments. Target-gene primer sequences are provided in Supplementary Table S3.

### 2.12. Western Blotting

Total cellular proteins were extracted using radioimmunoprecipitation assay (RIPA) buffer supplemented with protease and phosphatase inhibitor cocktails (GenDEPOT, Katy, TX, USA). Following incubation on ice, lysates were clarified by centrifugation at 13,000× *g* for 20 min at 4 °C, and protein concentrations were determined using the Pierce BCA Protein Assay Kit (catalog no. 23225; Thermo Fisher Scientific, Waltham, MA, USA). Equal amounts of protein were separated by SDS-PAGE and transferred onto polyvinylidene difluoride (PVDF) membranes (Millipore, Bedford, MA, USA). Membranes were blocked with 5% non-fat skim milk and incubated overnight at 4 °C with primary antibodies against phospho-NF-κB p65 (Ser536; catalog no. 3033), total NF-κB p65 (catalog no. 8242), phospho-p38 MAPK (Thr180/Tyr182; catalog no. 4511), total p38 MAPK (catalog no. 8690), phospho-JNK (Thr183/Tyr185; catalog no. 4668), total JNK (catalog no. 9252), phospho-ERK1/2 (Thr202/Tyr204; catalog no. 4370), total ERK1/2 (catalog no. 9102), phospho-Smad2 (Ser465/467; clone 138D4; catalog no. 3108), and total Smad2 (clone D43B4; catalog no. 5339), all from Cell Signaling Technology (Danvers, MA, USA). An anti-GAPDH antibody (catalog no. GTX100118; GeneTex, Irvine, CA, USA) was used as the loading control. A horseradish peroxidase (HRP)-conjugated goat anti-rabbit IgG (H+L) secondary antibody (catalog no. 7074S; Cell Signaling Technology, Danvers, MA, USA) was subsequently applied, and immunoreactive bands were visualized using enhanced chemiluminescence reagents (ECL Plus, Cytiva, Marlborough, MA, USA). Chemiluminescence images were acquired under identical exposure conditions and quantified using ImageJ software (version 1.54u; National Institutes of Health, Bethesda, MD, USA). The protein-normalization ratios are specified separately in the corresponding figure legends. Phosphorylated-protein signals were expressed relative to the corresponding total protein or GAPDH, as indicated in each plot. Ratios using different denominators were reported separately. Densitometric analyses were performed using at least three independent biological experiments.

### 2.13. Immunofluorescence Staining

L132 cells were fixed with 4% paraformaldehyde, permeabilized using 0.1% Triton X-100, and blocked with 1% bovine serum albumin in phosphate-buffered saline (PBS). Cells were incubated overnight at 4 °C with a mouse monoclonal antibody against collagen type I (clone COL-1; catalog no. sc-59772; Santa Cruz Biotechnology, Dallas, TX, USA) and rabbit antibodies against fibronectin (FN; catalog no. ab2413; Abcam, Cambridge, UK) and α-smooth muscle actin (α-SMA; clone D4K9N; catalog no. 19245S; Cell Signaling Technology, Danvers, MA, USA). After washing, cells were incubated with a fluorescein-conjugated horse anti-mouse IgG (H+L) secondary antibody for collagen type I detection (catalog no. FI-2000; Vector Laboratories, Newark, CA, USA) or an Alexa Fluor 488-conjugated donkey anti-rabbit IgG (H+L) secondary antibody for fibronectin and α-SMA detection (catalog no. A21206; Invitrogen, Waltham, MA, USA). The same primary antibody products were used for fibronectin and α-SMA detection in immunohistochemistry and immunofluorescence. Cells were mounted using VECTASHIELD Antifade Mounting Medium with DAPI (catalog no. H-1200; Vector Laboratories, Newark, CA, USA), which provided nuclear counterstaining. Fluorescence images were acquired using an Olympus BX53 fluorescence microscope (Olympus Corporation, Tokyo, Japan) under identical exposure settings for all treatment groups. Representative images were selected from at least three independent biological experiments.

### 2.14. Reproducibility and Replicates

Unless otherwise specified, all in vitro experiments were independently performed at least three times using separately prepared cell cultures. Technical replicates within each independent experiment were averaged prior to statistical analysis, such that biological replicates served as the unit of statistical inference. For the main in vivo experiments, each animal represented an independent biological replicate, with five mice per group. Independent animal cohorts were used for the 2-week and 6-week endpoints.

### 2.15. Statistical Analysis

Unless otherwise indicated, quantitative data are presented as the mean ± standard error of the mean (SEM). Unless otherwise indicated, all in vitro experiments were independently repeated at least three times, whereas each main in vivo experimental group consisted of five mice. Separate cohorts of mice were used for the 2-week (inflammatory phase) and 6-week (fibrotic phase) experiments. Main-study statistical analyses were performed using GraphPad Prism 9 (GraphPad Software, San Diego, CA, USA). Body weight measurements were analyzed using two-way repeated-measures analysis of variance (ANOVA). Cell viability experiments involving both radiation dose and incubation time were analyzed using two-way ANOVA. Comparisons among multiple groups at a single time point were performed using one-way ANOVA followed by Tukey’s post hoc multiple-comparison test. A *p* value < 0.05 was considered statistically significant.

For the main in vivo reference-treatment analyses, the reported Tukey-adjusted comparisons were no IR versus IR and IR versus each treatment group. Direct CYNC-1 versus prednisolone/nintedanib comparison results are not presented for these figures. Tukey’s multiple-comparison adjustment was applied to group comparisons within each endpoint. No additional multiplicity adjustment was applied across the full set of study endpoints. The HPLC values in Supplementary Table S2B represent single measurements from the 48 h storage assessment and are reported descriptively, without inferential comparisons or variability estimates. The independent preliminary micro-CT experiment in Figure S3 is summarized as mean ± sample standard deviation (SD; *n* = 4 mice per group). Total volume and damaged-region ratio are summarized descriptively where available. Inferential comparisons and the graph concern damaged-region volume in the five irradiated groups (20 mice). The non-irradiated control group was excluded from these comparisons because its damaged-region volumes were not reported. Ordinary one-way ANOVA with Tukey’s adjustment across all ten pairwise comparisons among these five groups was used; selected comparisons are displayed in the graph. Given the unequal observed SDs, Welch’s ANOVA with Games–Howell comparisons was also performed as a sensitivity analysis; both analyses are reported in Table S4C.

## 3. Results

### 3.1. Chromatographic Purity Profiles During Storage and Cellular Evaluation of CYNC-1

**Figure 1:**
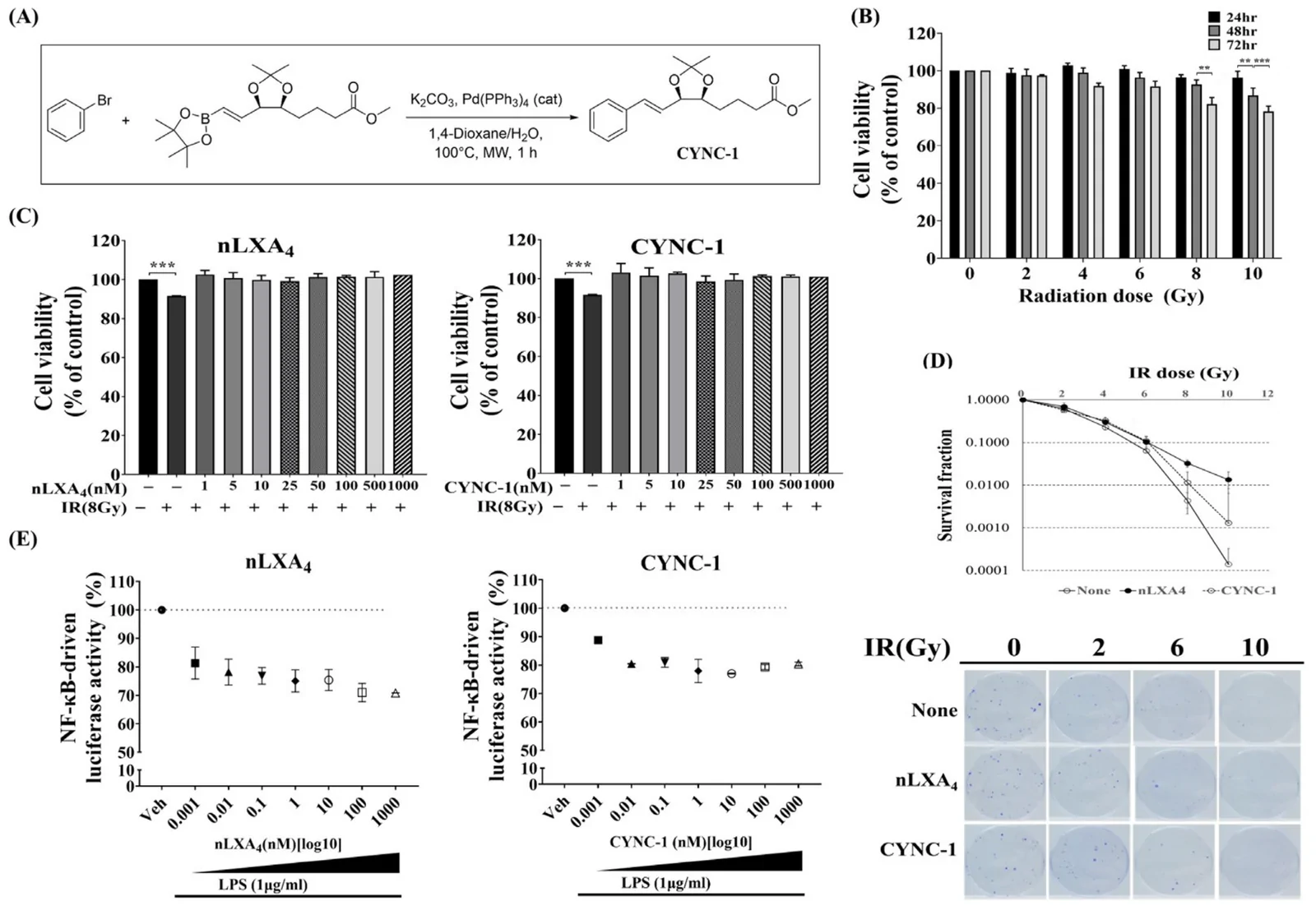
Synthesis and biological characterization of CYNC-1 in cell-based assays. (**A**) Molecular structure of CYNC-1; synthesis via Suzuki coupling reaction. (**B**) Cell viability in L132 cells after dose-dependent irradiation on days 1, 2, and 3. Cell viability was assessed using the WST-1 assay, with the results expressed relative to the control. Brackets indicate comparisons between the indicated post-irradiation time points at the same radiation dose. (**C**) L132 cells were pretreated with various concentrations of nLXA4 and CYNC-1 for 2 h, followed by 8 Gy irradiation and a 24 h incubation. The bar graphs show cell viability after irradiation in the presence of the indicated concentrations of nLXA4 or CYNC-1, relative to the non-irradiated control. The bracket indicates the comparison between the irradiation-only group and the non-irradiated control, both without nLXA4 or CYNC-1 treatment. ** *p* < 0.01, and *** *p* < 0.001. In panel (**C**), *** *p* < 0.001 versus the non-irradiated control without nLXA_4_ or CYNC-1 treatment. (**D**) Survival curves (upper) and representative images (lower) of the colony formation assay. Surviving fractions were calculated from colony counts with correction for plating efficiency, as described in Section 2.4. Cells were plated into 6-well plates, pretreated for 2 h with 1 nM nLXA4 or 1 nM CYNC-1, and irradiated with the indicated doses. Colonies were counted 14 days post-irradiation. Representative colony images are shown for 0, 2, 6, and 10 Gy; the survival curves show the indicated radiation doses. (**E**) NF-κB luciferase activity in THP1-Lucia™ NF-κB monocytes treated with the indicated concentrations of nLXA4 or CYNC-1. The cells were treated with nLXA4 or CYNC-1 for 2 h, followed by stimulation with either LPS (1000 ng/mL) or saline as the LPS solvent control for 24 h. The cell culture medium was subsequently collected and analyzed. All data are expressed as the mean ± SEM from three independent experiments.

CYNC-1 was designed and synthesized as an LXA4 mimetic based on structural modification of LXA4, with the aim of improving chemical stability while retaining biological activity in cell-based assays. CYNC-1 was prepared through a palladium-catalyzed Suzuki–Miyaura cross-coupling reaction using a boronic acid ester intermediate and bromobenzene, as illustrated in Figure 1A. The synthetic route, NMR characterization, and chromatographic measurements are described in Section 2.1. The NMR spectra and acquisition parameters are provided in Supplementary Figures S1 and S2 and Table S1; HPLC methods and storage measurements are presented together in Supplementary Table S2 and its accompanying methods.

In the storage assessment, CYNC-1 maintained high HPLC area purity in ethanol at −78, −20, and 4 °C over 48 h under light-protected conditions (Supplementary Table S2B). At 254 nm, the initial main-peak area percentages were 99.595% for CYNC-1 and 91.379% for nLXA4. At 4 °C after 48 h, the corresponding values were 98.441% and 87.097%; at −20 °C, they were 99.726% and 90.671%; and at −78 °C, they were 99.542% and 88.377%, respectively. Different starting concentrations and purities, compound-specific mobile-phase ratios and UV responses, and non-monotonic time-course values limit quantitative comparison between compounds. Each post-baseline value represents one measurement, with repeated sampling from the same vial within each sampling period. These area percentages do not quantify absolute recovery of intact compound or establish a degradation half-life.

### 3.2. CYNC-1 Preserves Survival of Irradiated L132 Cells and Reduces LPS-Induced NF-κB Reporter Activity

To determine the cellular activity of CYNC-1, we first established experimental conditions that produced reproducible radiation-induced L132 cell injury while maintaining sufficient viable cells for molecular analyses. Exposure of L132 cells to increasing radiation doses resulted in a dose- and time-dependent reduction in cell viability. An 8-Gy dose produced reproducible but non-lethal cytotoxicity over 72 h and was therefore selected for subsequent short-term viability experiments, whereas 6 Gy was used for molecular in vitro analyses requiring longer-term cell survival (Figure 1B).

Pretreatment with either native LXA4 (nLXA4) or CYNC-1 attenuated the radiation-associated reduction in L132 cell viability under the tested conditions (Figure 1C).

Clonogenic survival assays showed that pretreatment with nLXA4 or CYNC-1 increased the surviving fraction of irradiated L132 cells relative to irradiation alone (Figure 1D). This finding demonstrates cytoprotection in the tested HeLa-derived cell model and does not establish selective protection of normal lung epithelial cells.

In THP1-Lucia NF-κB reporter cells, LPS increased NF-κB-dependent luciferase activity, whereas pretreatment with nLXA4 or CYNC-1 reduced reporter activation (Figure 1E). These findings support anti-inflammatory activity in this assay.

### 3.3. Early CYNC-1 Administration Attenuates Radiation-Induced Lung Injury and Preserves Pulmonary Function In Vivo

**Figure 2:**
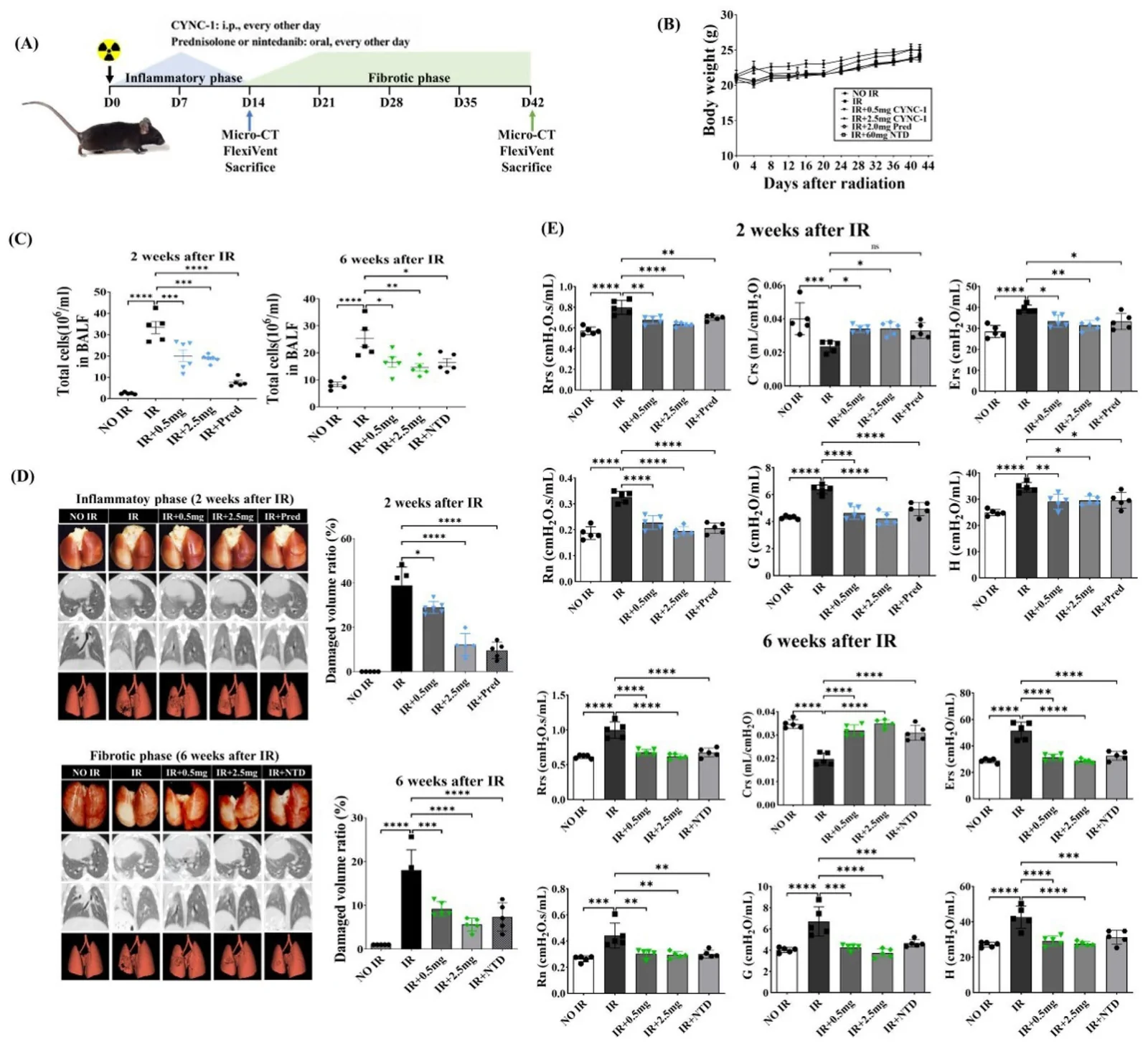
Effects of early CYNC-1 administration on lung injury assessed at 2 and 6 weeks after focal irradiation. (**A**) Experimental timeline. Mice received a single 75-Gy focal lung irradiation on day 0 (D0), indicated by the radiation symbol and downward arrow. All drug treatments began immediately after irradiation. CYNC-1 was administered intraperitoneally (i.p.) every other day for 2 or 6 weeks; prednisolone and nintedanib were administered orally every other day for 2 and 6 weeks, respectively. Independent cohorts were evaluated at the two endpoints. D denotes day relative to irradiation. The upward arrows at D14 and D42 indicate the respective assessment and sacrifice time points. (**B**) Body weight analysis of experimental groups: non-irradiated control (no IR), radiation-only group (IR), and groups treated with 0.5 or 2.5 mg/kg of CYNC-1 post-radiation. Prednisolone (Pred; 2 mg/kg) and nintedanib (NTD; 60 mg/kg) served as reference treatments in the 2- and 6-week cohorts, respectively. Throughout the figure, IR+0.5mg and IR+2.5mg denote irradiated mice treated with CYNC-1 at 0.5 and 2.5 mg/kg, respectively. IR+Pred and IR+NTD denote irradiated mice treated with prednisolone at 2 mg/kg and nintedanib at 60 mg/kg, respectively. Dose labels abbreviated as mg in the figure refer to doses in mg/kg body weight. (**C**) Quantification of total cell concentration in bronchoalveolar lavage fluid (BALF), expressed as 10^6^ cells/mL. BALF was collected via intratracheal instillation at 2 or 6 weeks post-irradiation. Cell pellets from centrifuged BALF samples were resuspended and analyzed using cytospin. Total cell counts were elevated in the radiation-only group, while treatment with 0.5 or 2.5 mg/kg CYNC-1, Pred, or NTD reduced cell counts compared to the radiation-only group. (**D**) Evaluation of radiation-associated lung injury using micro-CT. Representative gross findings (top row), axial and coronal micro-CT images (second and third rows), and three-dimensional reconstructions (bottom row) are shown for the non-irradiated control, irradiation-only, CYNC-1-treated, and corresponding reference-treatment groups. The accompanying graphs show the micro-CT-derived damaged lung volume ratio, expressed as a percentage, at 2 and 6 weeks after irradiation. (**E**) Lung function assessment using FlexiVent™ system. Parameters measured include Rrs, Crs, Ers, Rn, G, and H. All quantitative data are represented as the mean ± SEM, with *n* = 5 per group. Body-weight measurements (**B**) were analyzed using two-way repeated-measures ANOVA. Endpoint measurements in panels C–E were analyzed using one-way ANOVA followed by Tukey’s multiple-comparison test. Each bracket identifies the two groups being compared. The displayed comparisons are no IR versus IR and IR versus each treatment group. Direct CYNC-1 versus prednisolone/nintedanib comparison results are not presented. In the 2-week Crs plot, ns refers to IR versus IR+Pred. ns denotes an adjusted *p* value ≥ 0.05 for the indicated comparison and does not indicate equivalence between groups. * *p* < 0.05, ** *p* < 0.01, *** *p* < 0.001, **** *p* < 0.0001. Rrs, respiratory resistance; Crs, compliance of the respiratory system; Ers, elastance of the respiratory system; Rn, Newtonian resistance; G, tissue damping; H, tissue elastance. IR, irradiation; NO IR or no IR, non-irradiated control; SEM, standard error of the mean.

We next evaluated the effects of CYNC-1 in a focal mouse model of RILI at 2 and 6 weeks after irradiation. Treatment began immediately after irradiation and continued until the corresponding endpoint (Figure 2A).

No significant differences in body weight were observed among the experimental groups during the study (Figure 2B). This observation does not constitute a comprehensive tolerability or safety assessment.

Radiation exposure induced a marked inflammatory response, as reflected by a substantial increase in total bronchoalveolar lavage fluid (BALF) cellularity. Treatment with the synthetic mimetic significantly reduced inflammatory cell accumulation at both tested doses, with the higher dose producing the greatest reduction. Prednisolone was included as the reference treatment for the 2-week cohort (Figure 2C).

The reduction in inflammatory cellularity was accompanied by clear structural preservation of irradiated lung tissue. Micro-computed tomography (micro-CT) demonstrated extensive consolidation and loss of aerated lung volume following focal irradiation. These radiographic abnormalities were substantially attenuated by treatment with the synthetic mimetic at the 2- and 6-week endpoints. At the 6-week endpoint, mice receiving CYNC-1 from Day 0 showed less extensive radiographic lung injury. Representative images and quantitative measurements for the corresponding reference treatments are shown in Figure 2D.

To determine whether these structural improvements translated into functional benefit, respiratory mechanics were evaluated using the FlexiVent™ forced oscillation system. Focal irradiation significantly impaired both airway and parenchymal lung mechanics, as evidenced by increased respiratory resistance, elastance, tissue damping, and tissue elastance together with reduced respiratory compliance. Endpoint-specific results for CYNC-1 and the corresponding reference treatment are shown in Figure 2E. These data do not establish equivalence or non-inferiority between active treatments. At 2 weeks, the difference in respiratory system compliance (Crs) between the IR and IR+prednisolone groups was not statistically significant (Figure 2E).

Collectively, these findings demonstrate that the synthetic lipoxin mimetic attenuated both acute inflammatory injury and subsequent functional deterioration following focal lung irradiation, supporting further histopathological and molecular evaluation of the underlying tissue responses.

### 3.4. Histopathological Analyses Demonstrate Reduced Inflammatory Injury and Extracellular Matrix Remodeling

**Figure 3:**
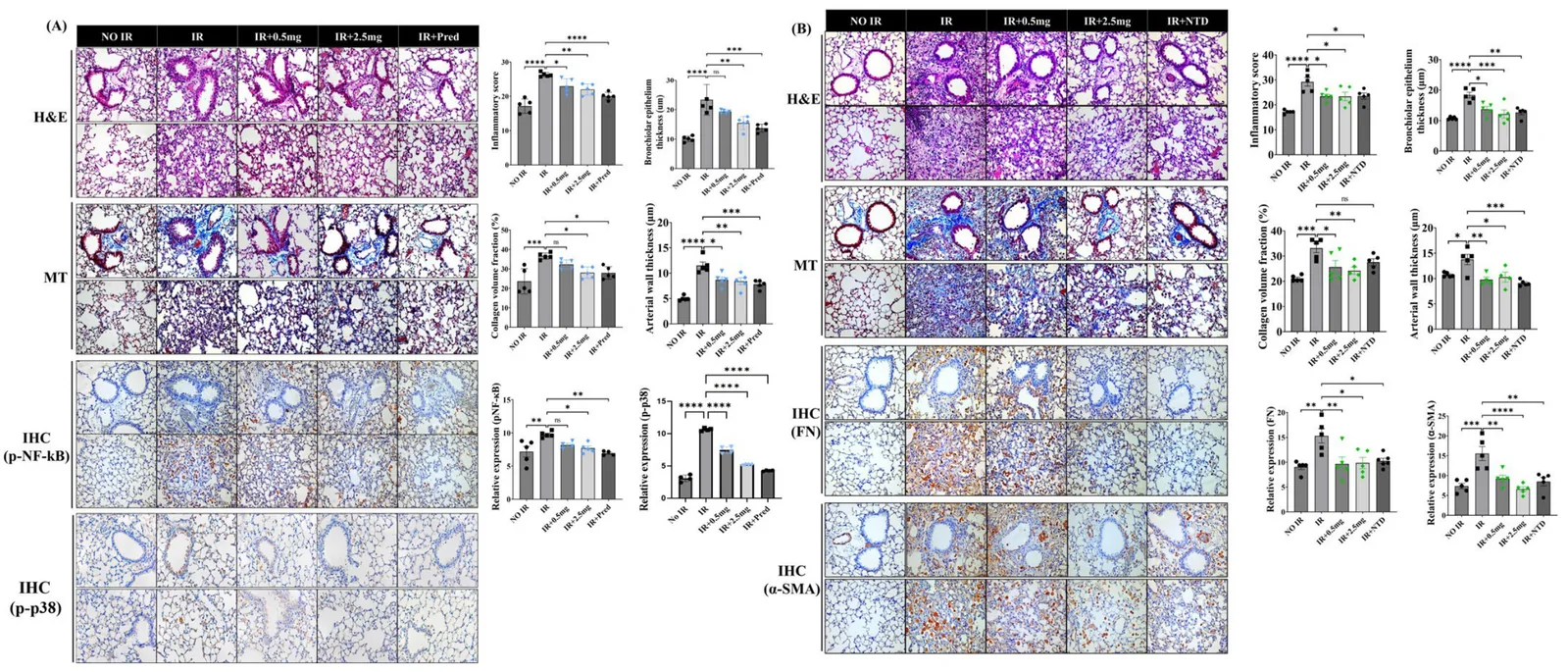
Pulmonary histopathology at 2 weeks (**A**) and 6 weeks (**B**) after focal irradiation in mice receiving treatment from Day 0. (**A**) Representative histological analysis of lung sections. From top to bottom, the image blocks show hematoxylin and eosin (H&E) staining, Masson’s trichrome (MT) staining, and immunohistochemistry for p-NF-κB p65 and p-p38. Experimental groups included non-irradiated control (no IR), radiation-only group (IR, 75 Gy), groups treated with 0.5 or 2.5 mg/kg of CYNC-1 post-radiation, and group treated with 2 mg/kg of Pred post-radiation. Scale bar = 50 μm. Quantitative bar graphs illustrate inflammatory changes in lung tissues by measuring parameters such as inflammatory score, bronchiolar epithelial thickness, collagen volume fraction, arterial wall thickness, and relative expression of p-NF-κB p65 and p-p38. (**B**) Histological evaluation of fibrosis-related markers. From top to bottom, the image blocks show H&E staining, MT staining, and immunohistochemistry for fibronectin and α-SMA. Experimental groups consisted of non-irradiated control (no IR), radiation-only group (IR, 75 Gy), groups treated with 0.5 or 2.5 mg/kg of CYNC-1 post-radiation, and group treated with 60 mg/kg of NTD post-radiation. Scale bar = 50 μm. Bar graphs present the quantitative analysis of lung tissue remodeling, including measurement of inflammatory score, bronchiolar epithelial thickness, collagen volume fraction, arterial wall thickness, and relative expression of FN and α-SMA across treatment groups. All data are represented as the mean ± SEM, with *n* = 5 per group. Endpoint measurements were analyzed using one-way ANOVA followed by Tukey’s multiple-comparison test. The displayed comparisons are no IR versus IR and IR versus each treatment group. Direct CYNC-1 versus prednisolone/nintedanib comparison results are not presented. * *p* < 0.05, ** *p* < 0.01, *** *p* < 0.001, **** *p* < 0.0001, ns, not significant.

Histopathological analyses were performed to determine whether the structural and functional improvements observed in vivo were accompanied by attenuation of tissue injury. During the inflammatory phase, hematoxylin and eosin (H&E) staining demonstrated extensive inflammatory cell infiltration, bronchiolar epithelial thickening, and vascular remodeling in irradiated lungs. These pathological changes were substantially reduced following treatment with the synthetic lipoxin mimetic, particularly at the higher dose, and were accompanied by lower histological inflammation scores and reduced bronchiolar epithelial and arterial wall thicknesses (Figure 3A).

Masson’s trichrome staining further demonstrated that irradiation induced early extracellular matrix deposition within the irradiated lung, whereas treatment markedly reduced collagen accumulation. Immunohistochemical analyses showed corresponding reductions in phosphorylated NF-κB p65 and phosphorylated p38 MAPK, consistent with attenuation of radiation-induced inflammatory signaling at the tissue level (Figure 3A).

At the 6-week endpoint, mice receiving CYNC-1 from Day 0 showed less collagen deposition and architectural distortion than irradiated vehicle-treated mice (Figure 3B). Fibronectin and α-SMA staining was also reduced, consistent with lower expression of fibrosis-associated markers. These observations document attenuation of fibrotic remodeling that developed after irradiation during continued treatment.

Quantitative morphometric results are presented for each endpoint and dose in Figure 3A,B. The magnitude and statistical significance of the treatment-associated changes varied across endpoints and doses. Taken together, the histological and morphometric findings support attenuation of inflammatory injury and subsequent fibrotic remodeling after early CYNC-1 administration.

### 3.5. CYNC-1 Treatment Is Associated with Reduced Inflammatory Cytokine Expression and NF-κB/MAPK Phosphorylation

**Figure 4:**
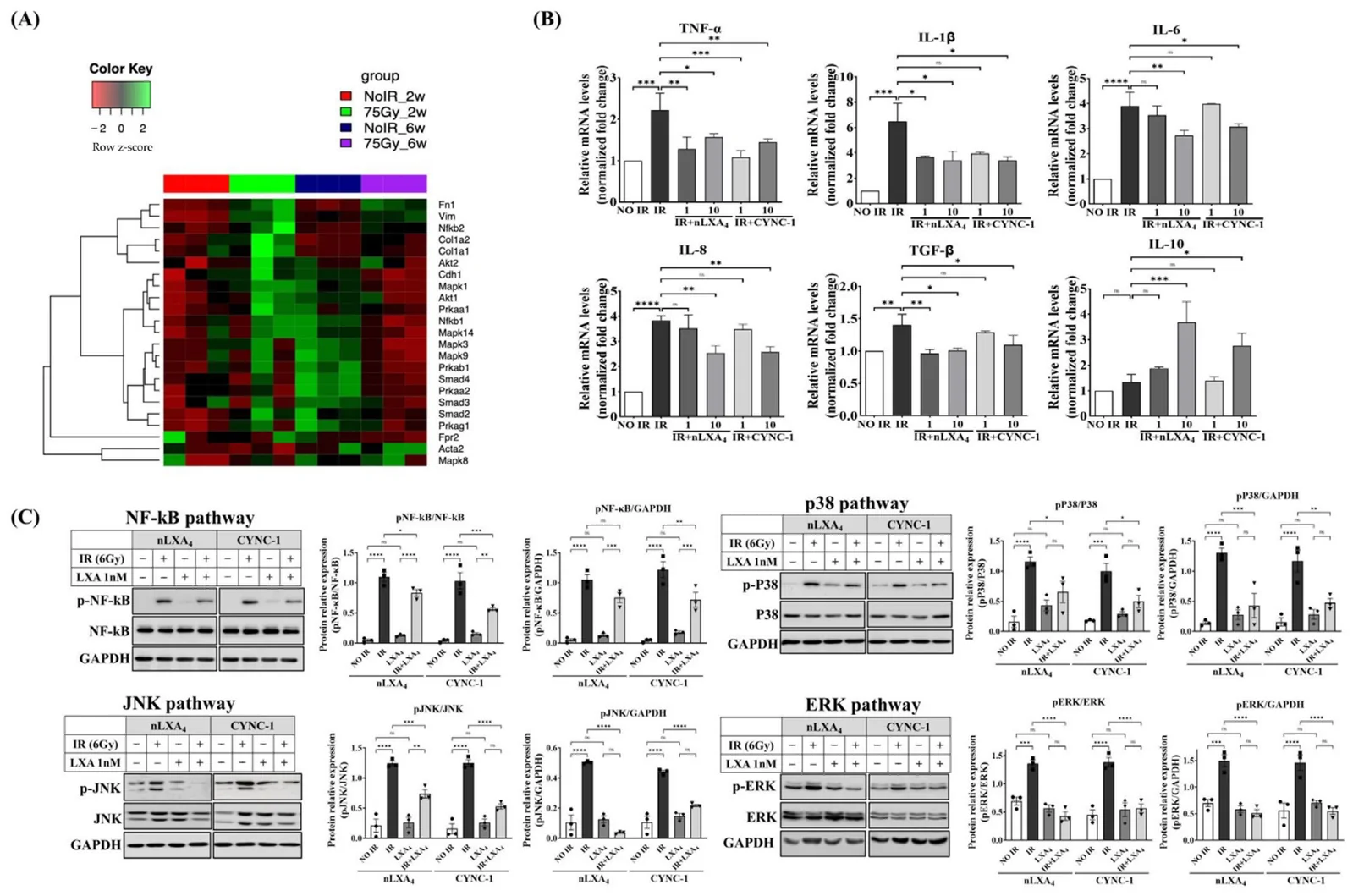
Phase-specific gene expression in irradiated mouse lung tissue and the effects of CYNC-1 on inflammatory signaling in L132 cells. (**A**) Heatmap of 23 selected inflammation- and fibrosis-associated genes from the previously published RNA-seq dataset GSE205025 [40]. The four groups comprise non-irradiated controls and 75-Gy-irradiated mouse lung samples collected at 2 and 6 weeks. In the group labels, NoIR indicates non-irradiated controls, 75Gy indicates 75-Gy irradiation, and 2w and 6w indicate the respective sampling times in weeks. Each column represents RNA from one independent mouse (*n* = 3 mice per group; 12 samples in total), without pooling. Colors indicate row z-scores of read counts across all 12 samples, from red (below the gene-specific mean) through black (near the mean) to green (above the mean). The row dendrogram represents hierarchical clustering using Pearson correlation distance and average linkage. This panel provides radiation-model context and does not assess CYNC-1 treatment effects. (**B**) Quantitative RT-qPCR analysis of cytokine mRNA levels in L132 cells, including the inflammatory cytokines TNF, IL1B, IL6, and CXCL8, the profibrotic cytokine TGFB1, and the anti-inflammatory cytokine IL10. These are labeled TNF-α, IL-1β, IL-6, IL-8, TGF-β, and IL-10, respectively, in the plots; the TGF-β plot specifically represents TGFB1 mRNA. Cells were pretreated with 1 and 10 nM nLXA4 or CYNC-1 for 2 h, followed by 6 Gy irradiation and incubation for 24 h. Total RNA was isolated 24 h post-radiation, and RT-qPCR was performed. Expressions of cytokine mRNA were calculated relative to the non-irradiated control and normalized against GAPDH. The values 1 and 10 beneath the treatment groups indicate compound concentrations in nM. (**C**) Expressions of p-NF-κB p65, p-p38, p-JNK, and p-ERK in L132 cells assessed using Western blot analysis in NF-κB and MAPK pathway activation. Cells were pretreated with 1 nM of nLXA4 or CYNC-1 for 2 h, followed by 6 Gy irradiation and a 24 h incubation. The NF-κB plots show p-NF-κB p65/total NF-κB p65 and p-NF-κB p65/GAPDH ratios. For p38, JNK, and ERK, phosphorylated/total-protein and phosphorylated-protein/GAPDH ratios are shown separately. Phosphorylated-protein signals were expressed relative to the corresponding total protein or GAPDH, as indicated in each plot. The NF-κB and p-NF-κB labels in this panel refer to total and phosphorylated NF-κB p65, respectively. The abbreviation nLXA denotes nLXA4. In the treatment tables and plot labels, LXA denotes the compound identified by the corresponding heading: nLXA4 in the nLXA/nLXA4 sections and CYNC-1 in the CYNC-1 sections. Thus, IR+LXA indicates irradiation combined with the respective compound, and the LXA 1nM row indicates treatment with that compound at 1 nM. The “+” and “−” symbols indicate the presence and absence, respectively, of irradiation or the indicated compound treatment. Quantitative data in panels B and C are represented as mean ± SEM from three independent experiments. Statistical comparisons are indicated by brackets: * *p* < 0.05, ** *p* < 0.01, *** *p* < 0.001, **** *p* < 0.0001, ns, not significant. IR, irradiation; No IR or NO IR, non-irradiated control; nLXA4, native lipoxin A4; p-, phosphorylated; NF-κB, nuclear factor kappa B; MAPK, mitogen-activated protein kinase; JNK, c-Jun N-terminal kinase; ERK, extracellular signal-regulated kinase; GAPDH, glyceraldehyde-3-phosphate dehydrogenase; SEM, standard error of the mean.

To characterize radiation-associated molecular changes, we reanalyzed the published GSE205025 dataset from Farh et al. [40], comparing 75-Gy-irradiated and time-matched non-irradiated mouse lungs. The heatmap displays row-standardized expression patterns among selected inflammatory-signaling and matrix-associated genes at 2 and 6 weeks (Figure 4A). This analysis provides context for the radiation-injury model and does not test a CYNC-1 treatment effect or establish pathway enrichment from the selected genes alone.

We next measured cytokine expression in irradiated L132 cells to examine the cellular response to CYNC-1. Irradiation increased expression of the inflammatory cytokines TNF, IL1B, IL6, and CXCL8 and the profibrotic cytokine TGFB1 (Figure 4B). CYNC-1 treatment was associated with lower expression of several inflammatory or profibrotic transcripts and higher IL10 expression under some of the tested conditions. The responses varied by transcript and concentration (Figure 4B). The nLXA4 results are shown alongside those for CYNC-1; phenotypic similarities between these compounds do not establish a shared receptor mechanism.

Irradiation increased phosphorylation of NF-κB p65, p38, JNK, and ERK in L132 cells. Pretreatment with CYNC-1 reduced phosphorylation of these proteins under the tested conditions. Corresponding results for nLXA4 are shown in Figure 4C. These observations identify signaling changes associated with treatment but do not establish whether these pathways are required for the observed effects.

The cellular findings show that CYNC-1 treatment was associated with reduced inflammatory cytokine expression and lower NF-κB/MAPK phosphorylation in L132 cells.

### 3.6. CYNC-1 Treatment Is Associated with Reduced Profibrotic Marker Expression and Smad2 Phosphorylation in L132 Cells

**Figure 5:**
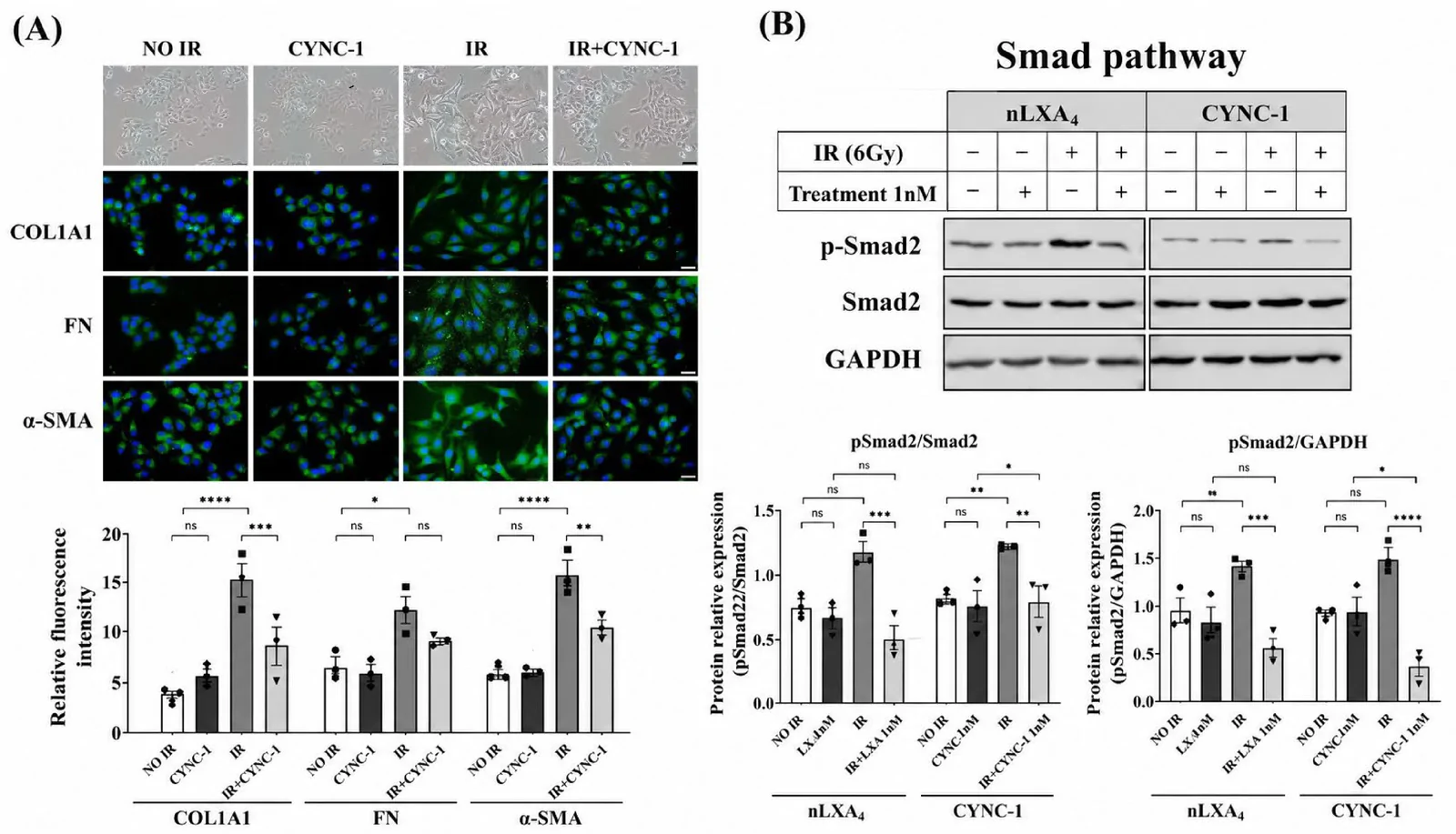
Effects of CYNC-1 on radiation-associated morphological changes, profibrotic marker expression, and Smad2 phosphorylation in L132 cells. (**A**) Cell morphology and profibrotic marker expression. L132 cells were pretreated with or without 1 nM CYNC-1 for 2 h before 6-Gy irradiation or no irradiation, and morphology and expression of COL1A1, fibronectin, and α-SMA were assessed at 48 h post-treatment. Representative images of cell morphology are shown. The morphology images illustrate changes in cell shape; the accompanying bar graphs quantify the relative fluorescence intensity of COL1A1, fibronectin, and α-SMA. Scale bar = 50 μm. Representative immunofluorescence images are shown in the second, third, and bottom rows. Green fluorescence indicates immunostaining for the indicated target protein, and blue fluorescence indicates DAPI-stained nuclei. (**B**) Western blot analysis of Smad2 phosphorylation. L132 cells were pretreated with 1 nM nLXA4 or CYNC-1 for 2 h, irradiated with 6 Gy, and harvested 48 h later for Western blotting. The p-Smad2/Smad2 and p-Smad2/GAPDH ratios were calculated separately. All data are presented as mean ± SEM from three independent experiments. * *p* < 0.05, ** *p* < 0.01, *** *p* < 0.001, **** *p* < 0.0001, ns, not significant.

We next examined radiation-associated morphological changes and profibrotic markers in L132 cells. Representative images showed cellular elongation and cytoplasmic extensions after irradiation, with less apparent morphological alteration in CYNC-1-pretreated cells (Figure 5A).

Irradiation increased COL1A1, fibronectin, and α-SMA immunofluorescence. CYNC-1 pretreatment significantly reduced COL1A1 and α-SMA immunofluorescence relative to irradiation alone, whereas the reduction in fibronectin did not reach statistical significance (Figure 5A). These findings indicate a radiation-associated profibrotic phenotype with marker-dependent responses to CYNC-1 in this cell model.

Western blot analysis showed increased Smad2 phosphorylation after irradiation and lower phosphorylation in L132 cells pretreated with CYNC-1 or nLXA4 (Figure 5B). These observations identify treatment-associated changes in Smad2 phosphorylation.

Together, these data show that CYNC-1 treatment was associated with reduced radiation-associated morphological changes, profibrotic marker expression, and Smad2 phosphorylation in L132 cells.

## 4. Discussion

The present study shows that CYNC-1 treatment initiated immediately after irradiation attenuated early inflammatory injury and subsequent fibrotic remodeling in a focal mouse model of RILI. CYNC-1 also maintained high HPLC area purity during 48 h of light-protected ethanol storage at −78, −20, and 4 °C and showed activity in cellular radiation-response and inflammatory assays. In L132 cells, treatment was associated with lower NF-κB/MAPK and Smad2 phosphorylation and reduced profibrotic marker expression. These findings support further preclinical investigation of CYNC-1. Their interpretation requires consideration of the early treatment schedule, the associative nature of the signaling findings, the unverified receptor pharmacology, and the need to preserve antitumor efficacy.

Despite the well-recognized anti-inflammatory and pro-resolving properties of native LXA4, its therapeutic translation has been hindered by rapid metabolic inactivation, poor chemical stability, and a short biological half-life [25,32,33]. To overcome these limitations, successive generations of synthetic LXA4 mimetics have been developed with the aim of improving chemical and metabolic stability, receptor activity, and other pharmacological properties. Building upon these previous efforts, CYNC-1 was rationally designed through structural modifications intended to enhance resistance to degradation while preserving LXA4-like biological activity [32,33,34,35,36,37]. CYNC-1 retained high HPLC area purity during 48 h of light-protected ethanol storage at −78, −20, and 4 °C and showed cytoprotective and anti-inflammatory activity in the assays performed. Both compounds were evaluated at 254 nm, but different starting concentrations and purities, compound-specific mobile-phase ratios and UV responses, and non-monotonic values limit quantitative comparison of degradation with nLXA4. The storage assessment used single measurements and repeated vial sampling, which did not provide independent replicate samples. Storage in light-protective containers in unbuffered ethanol does not establish photostability or controlled-pH stability. Main-peak area percentages do not establish absolute compound recovery, degradation half-life, metabolic stability, or systemic exposure.

Our previous study investigated a structurally distinct synthetic LXA4 analogue in RILI [39]. The current study extends this work by evaluating CYNC-1-associated changes in NF-κB/MAPK and Smad2 signaling alongside inflammatory and fibrotic endpoints. Pharmacokinetic studies and direct receptor pharmacology are needed to relate the chemical modifications to exposure and biological activity.

CYNC-1 treatment initiated on Day 0 was associated with lower inflammatory injury at 2 weeks and reduced fibrotic remodeling at 6 weeks. Because treatment began before fibrosis developed and continued throughout follow-up, the 6-week findings demonstrate attenuation of subsequent fibrotic remodeling rather than reversal or treatment of established fibrosis. The present design cannot distinguish a direct effect on fibrogenesis from the downstream consequences of reduced early radiation injury. A delayed-treatment study initiated after fibrosis has been documented is needed to assess efficacy against established pulmonary fibrosis.

Prednisolone and nintedanib served as reference treatments in the respective experiments. The comparisons presented do not establish equivalent or non-inferior efficacy of CYNC-1 relative to either drug. Statistical significance versus IR in separate treatment groups does not establish similarity between those treatments; a non-significant direct comparison would not, by itself, demonstrate equivalence or non-inferiority. CYNC-1 was administered intraperitoneally, whereas the reference drugs were given orally; differences in absorption, bioavailability, and systemic exposure may therefore affect the observed responses. Without exposure-matched comparisons and pharmacokinetic data, relative pharmacological potency cannot be determined from these regimens.

In L132 cells, CYNC-1 treatment was associated with reduced phosphorylation of NF-κB p65, p38, JNK, ERK, and Smad2, together with lower inflammatory cytokine and profibrotic marker expression. These parallel changes are consistent with modulation of inflammatory and profibrotic signaling. However, pathway inhibition, activation, genetic perturbation, and rescue experiments were not performed to establish whether these pathways are required for the observed effects. The measurements also do not establish direct regulation of the TGF-β receptor. Reduced pathway activation could be secondary to reduced cellular injury rather than direct pathway targeting. The molecular observations in L132 cells therefore represent treatment-associated changes and do not establish a causal mechanism for the attenuation of lung injury or fibrotic remodeling observed in mice.

**Figure 6:**
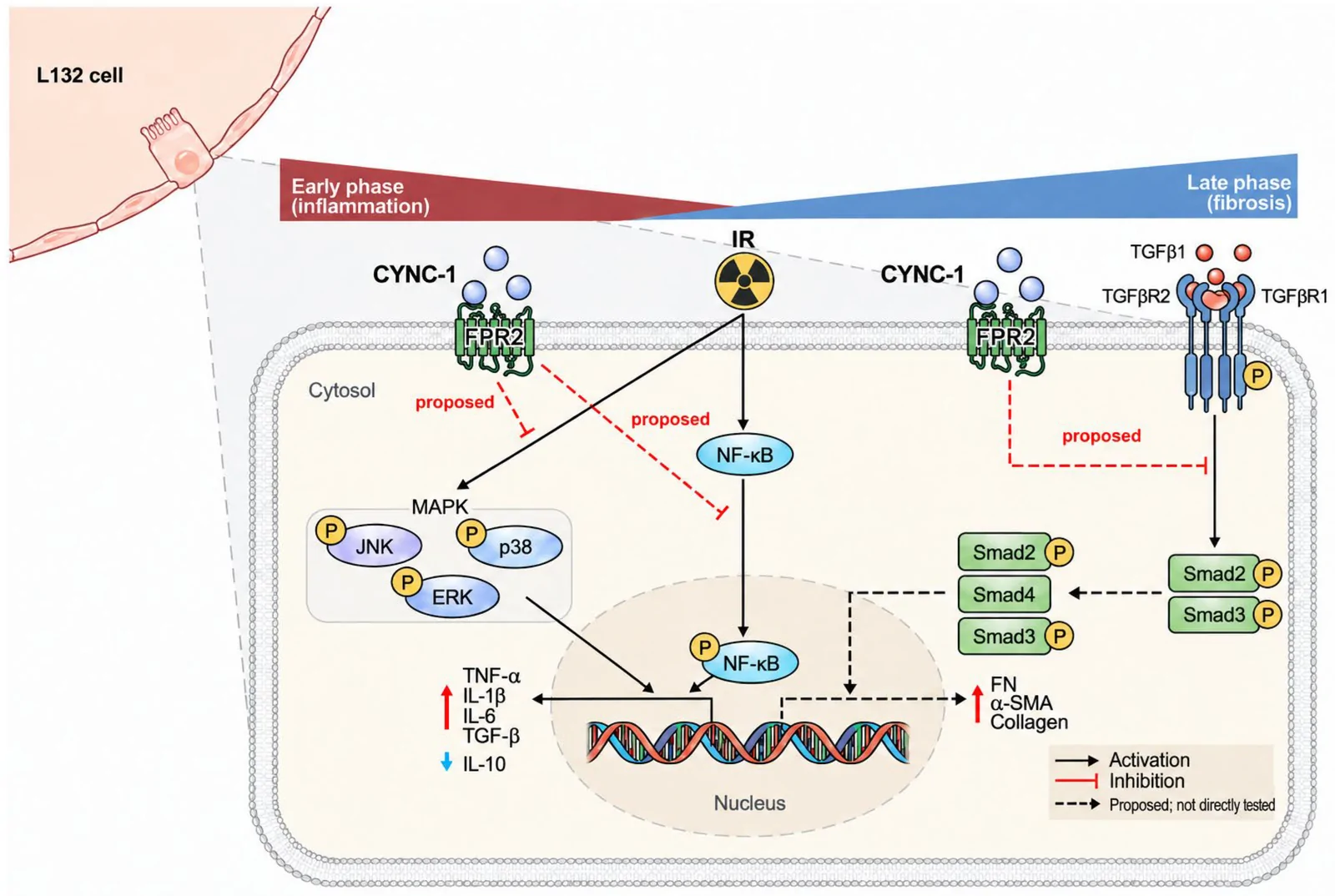
Proposed signaling model contextualizing the effects of CYNC-1 in RILI. In L132 cells, CYNC-1 treatment was associated with reduced phosphorylation of NF-κB p65, p38, JNK, ERK, and Smad2 and changes in inflammatory and profibrotic marker expression. Solid arrows provide signaling context; dashed arrows and inhibitory connections depict proposed relationships that were not established by the present experiments. Red upward and blue downward arrows depict schematic increases and decreases, respectively. Direct CYNC-1 binding to FPR2, receptor dependence, direct TGF-β receptor regulation, and involvement of Smad3/Smad4 were not established. The cytokine and matrix-marker arrows illustrate a schematic inflammatory/profibrotic state, not the direction of CYNC-1 treatment effects. Measured cytokine responses, including higher IL10 expression under some treatment conditions, varied by transcript and concentration (Figure 4B). The early- and late-phase labels provide a conceptual framework for RILI; treatment in the mouse experiments began on Day 0 and did not test treatment of established fibrosis.

CYNC-1 was designed as an LXA4 mimetic based on structural modification of LXA4, and the phenotypic similarities to nLXA4 observed in the present assays are consistent with this design rationale. Here, the term “mimetic” denotes the design rationale and observed LXA4-like phenotypic effects, not a pharmacologically validated FPR2 agonist. Direct FPR2 engagement, receptor-selective agonism, and FPR2 dependence were not established in this study. Similar suppression of NF-κB/MAPK signaling does not demonstrate a shared receptor mechanism. Canonical LXA4 receptor signaling provides a biological rationale for the proposed involvement of FPR2 [27,28,31,41]. Direct receptor pharmacology and studies combining FPR2 blockade or genetic loss of function with functional endpoints are needed to test this hypothesis. Figure 6 presents this receptor-level hypothesis within a proposed signaling model; the depicted connections do not establish mechanisms of CYNC-1 action.

Specialized pro-resolving mediators provide a rationale for investigating tissue protection [23,24,25,32,42,43,44]. The in vivo findings support further investigation of CYNC-1 for attenuation of radiation-associated lung injury. Its development as a normal-tissue protectant during radiotherapy, however, requires preservation of antitumor efficacy. The increased clonogenic survival observed in HeLa-derived L132 cells demonstrates cytoprotection in the tested model but does not establish selective protection of normal lung epithelium and leaves open the possibility of protection of malignant cells. Dedicated studies measuring clonogenic radiation responses in relevant tumor cell models and tumor growth delay or local control in tumor-bearing animals are needed to determine whether CYNC-1 preserves antitumor efficacy. The current study therefore does not establish an improvement in the therapeutic ratio.

The cellular findings are limited by the use of HeLa-derived L132 cells and require validation in lung-relevant, non-transformed cell systems. Validation in primary human alveolar epithelial cells and non-transformed pulmonary cell models, preferably from multiple donors, is needed to assess lung-specific relevance. Relevant endpoints include radiation-associated cell injury, epithelial barrier function where applicable, cytokine responses, and the signaling changes examined in the present study. Complementary studies in primary human lung fibroblasts could assess matrix production and fibroblast activation. In L132 cells, the observed morphological changes and increased COL1A1, fibronectin, and α-SMA expression are consistent with a profibrotic phenotype but do not establish EMT, because loss of epithelial markers, such as E-cadherin, and broader changes in epithelial identity were not evaluated. These cellular findings also do not establish a contribution of epithelial EMT to pulmonary fibrosis in vivo.

The animal study used a single 75-Gy exposure to a small region of the left lung. The dose, irradiated volume, and temporal pattern differ from those of conventionally fractionated thoracic radiotherapy, limiting direct extrapolation to clinical treatment. Evaluation under clinically relevant fractionated irradiation schedules is needed to assess whether the observed protection extends to those settings. Because CYNC-1 administration began on Day 0, a separate delayed-treatment design is also needed to evaluate efficacy after fibrosis has developed.

Only male mice were studied, and sex-dependent differences in radiation-induced lung injury or the response to CYNC-1 were not assessed. The present findings therefore cannot be assumed to apply equally to females. Studies including both sexes are needed to evaluate the generalizability of the findings.

The study evaluated multiple endpoints in relatively small experimental groups. Tukey’s adjustment was applied to group comparisons within each endpoint, but no additional multiplicity adjustment was applied across the full set of endpoints. Study-wide control of the family-wise error rate therefore cannot be assumed. The endpoint-wise statistical findings should be interpreted as exploratory and require confirmation in appropriately designed studies. The independent dose-selection experiment was small (*n* = 4 per group), and the observed SDs differed among groups. The key dose-selection comparisons were consistent in the unequal-variance sensitivity analysis. Comparisons of IR with CYNC-1 at 2.5 or 5 mg/kg and with prednisolone were significant in both analyses, whereas the IR-versus-CYNC-1 0.5 mg/kg comparison and comparisons of CYNC-1 at 2.5 or 5 mg/kg with prednisolone were sensitive to the analytical method (Supplementary Table S4C). The apparent plateau between 2.5 and 5 mg/kg requires confirmation in a dedicated dose–response study; a non-significant difference does not demonstrate dose equivalence or pharmacological saturation.

Additional pharmacokinetic and pharmacodynamic studies are needed, together with evaluation of immune-cell populations and resolution-associated responses during RILI [24,25,26,42,45].

## 5. Conclusions

CYNC-1 maintained high HPLC area purity during light-protected ethanol storage for 48 h at −78, −20, and 4 °C and attenuated lung inflammation and subsequent fibrotic remodeling when administered from Day 0 in a focal irradiation model using male mice. In L132 cells, treatment was associated with reduced NF-κB/MAPK and Smad2 phosphorylation and lower profibrotic marker expression. These findings support further preclinical investigation, while FPR2 dependence, causal pathway involvement, and efficacy against established pulmonary fibrosis remain to be established.

Extrapolation to clinical radiotherapy is limited by the single high-dose irradiation model and the absence of validation in primary human lung cells. Preservation of antitumor efficacy remains an essential unresolved requirement, particularly in view of the cytoprotection observed in HeLa-derived L132 cells. Further evaluation using clinically relevant fractionated irradiation models, animals of both sexes, and tumor-bearing models will be necessary to determine whether CYNC-1 can be developed as a normal-tissue protectant during radiotherapy.

## Data Availability

The results reported in this study are presented in the article and its Supplementary Materials. The previously published RNA-seq dataset reanalyzed for Figure 4A is publicly available in GEO under accession GSE205025 (https://www.ncbi.nlm.nih.gov/geo/query/acc.cgi?acc=GSE205025, accessed on 17 October 2026), as reported by Farh et al. [40]. Raw sequencing reads are available through the linked Sequence Read Archive records, and the gene-count matrix is available as GSE205025_raw_gene_count.txt.gz on the GEO series record. The sample subset used for the present analysis is specified in Section 2.10. Individual measurements and statistical comparisons from the independent preliminary micro-CT experiment are provided in Supplementary S4.

## Supplementary Materials

The following supporting information can be downloaded at: https://www.mdpi.com/article/10.3390/pharmaceutics18101321/s1, Figure S1: ^1^H NMR spectrum of CYNC-1 recorded at 400.035 MHz in DMSO at 25.0 °C; Figure S2: ^13^C NMR spectrum of CYNC-1 recorded at 100.600 MHz in DMSO at 25.0 °C; Table S1: NMR acquisition parameters for CYNC-1; Table S2: HPLC analytical conditions and ethanol-storage assessment—(A) analytical conditions and (B) main-peak area percentages at 254 nm over 48 h at −78, −20, and 4 °C, with accompanying preparation and sampling methods; Table S3: Target gene primers for RT-qPCR; Figure S3: Exploratory dose-ranging evaluation of CYNC-1 after lung irradiation—(A) representative micro-CT images obtained two weeks after irradiation and (B) quantification of damaged-region volume; Table S4: (A) Group summary; (B) Individual micro-CT measurements; (C) Statistical comparisons of damaged-region volume.

## Abbreviations

The following abbreviations are used in this manuscript:

α-SMA	Alpha-Smooth Muscle Actin
ALX/FPR2	Lipoxin A4 receptor/formyl peptide receptor 2
BALF	Bronchoalveolar Lavage Fluid
COL1A1	Collagen Type I Alpha 1 Chain
CT	Computed Tomography
Crs	Compliance of the Respiratory System
DMEM	Dulbecco’s Modified Eagle’s Medium
ECM	Extracellular Matrix
EMT	Epithelial–Mesenchymal Transition
ERK	Extracellular Signal-Regulated Kinase
Ers	Elastance of the Respiratory System
FBS	Fetal Bovine Serum
FN	Fibronectin
FOT	Forced Oscillation Technique
G	Tissue Damping
GAPDH	Glyceraldehyde 3-Phosphate Dehydrogenase
H	Tissue Elastance
H&E	Hematoxylin and Eosin
HPLC	High-Performance Liquid Chromatography
IL	Interleukin
IR	Ionizing Radiation
JNK	c-Jun N-terminal kinase
LPS	Lipopolysaccharide
LXA4	Lipoxin A4
MAPK	Mitogen-Activated Protein Kinase
MT	Masson’s Trichrome
nLXA4	Native LXA4
NF-κB	Nuclear Factor kappa-light-chain-enhancer of activated B cells
NTD	Nintedanib
PBS	Phosphate-Buffered Saline
p-Smad2	Phosphorylated Smad2
Pred	Prednisolone
RILI	Radiation-Induced Lung Injury
Rn	Newtonian Resistance
ROS	Reactive Oxygen Species
RT	Radiotherapy
RT-qPCR	Quantitative Real-Time Polymerase Chain Reaction
Rrs	Respiratory System Resistance
SEM	Standard Error of the Mean
TGF-β	Transforming Growth Factor Beta
TNF-α	Tumor Necrosis Factor Alpha

## References

[1] Giuranno L., Ient J., De Ruysscher D., Vooijs M.A. (2019). Radiation-induced lung injury (RILI). Front. Oncol..

[2] Kuipers M.E., van Doorn-Wink K.C.J., Hiemstra P.S., Slats A.M. (2024). Predicting radiation-induced lung injury in patients with lung cancer: Challenges and opportunities. Int. J. Radiat. Oncol. Biol. Phys..

[3] Roy S., Salerno K.E., Citrin D.E. (2021). Biology of radiation-induced lung injury. Semin. Radiat. Oncol..

[4] Hanania A.N., Mainwaring W., Ghebre Y.T., Hanania N.A., Ludwig M. (2019). Radiation-induced lung injury: Assessment and management. Chest.

[5] Dy G.K., Prasad D., Kumar P., Attwood K., Adjei A.A. (2021). A phase 2 randomized, double-blind, placebo-controlled study evaluating nintedanib versus placebo as prophylaxis against radiation pneumonitis in patients with unresectable NSCLC undergoing chemoradiation therapy. J. Thorac. Oncol..

[6] Rimner A., Moore Z.R., Lobaugh S., Geyer A., Gelblum D.Y., Abdulnour R.E.E., Shepherd A.F., Shaverdian N., Wu A.J., Cuaron J. (2023). Randomized phase 2 placebo-controlled trial of nintedanib for the treatment of radiation pneumonitis. Int. J. Radiat. Oncol. Biol. Phys..

[7] Jin H., Yoo Y., Kim Y., Kim Y., Cho J., Lee Y.S. (2020). Radiation-induced lung fibrosis: Preclinical animal models and therapeutic strategies. Cancers.

[8] Huang Y., Zhang W., Yu F., Gao F. (2017). The cellular and molecular mechanism of radiation-induced lung injury. Med. Sci. Monit..

[9] Citrin D.E., Mitchell J.B. (2017). Mechanisms of normal tissue injury from irradiation. Semin. Radiat. Oncol..

[10] Siva S., MacManus M., Kron T., Best N., Smith J., Lobachevsky P., Ball D., Martin O. (2014). A pattern of early radiation-induced inflammatory cytokine expression is associated with lung toxicity in patients with non-small cell lung cancer. PLoS ONE.

[11] Liu X., Shao C., Fu J. (2021). Promising biomarkers of radiation-induced lung injury: A review. Biomedicines.

[12] Liu T., Zhang L., Joo D., Sun S.C. (2017). NF-κB signaling in inflammation. Signal Transduct. Target. Ther..

[13] Sarkar F.H., Li Y., Wang Z., Kong D. (2008). NF-κB signaling pathway and its therapeutic implications in human diseases. Int. Rev. Immunol..

[14] Shi J.H., Sun S.C. (2018). Tumor necrosis factor receptor-associated factor regulation of nuclear factor-κB and mitogen-activated protein kinase pathways. Front. Immunol..

[15] Schulze-Osthoff K., Ferrari D., Riehemann K., Wesselborg S. (1997). Regulation of NF-κB activation by MAP kinase cascades. Immunobiology.

[16] Ding N.H., Li J.J., Sun L.Q. (2013). Molecular mechanisms and treatment of radiation-induced lung fibrosis. Curr. Drug Targets.

[17] Wynn T.A., Ramalingam T.R. (2012). Mechanisms of fibrosis: Therapeutic translation for fibrotic disease. Nat. Med..

[18] Flanders K.C. (2004). Smad3 as a mediator of the fibrotic response. Int. J. Exp. Pathol..

[19] Roberts A.B., Russo A., Felici A., Flanders K.C. (2003). Smad3: A key player in pathogenetic mechanisms dependent on TGF-β. Ann. N. Y. Acad. Sci..

[20] Kalluri R., Weinberg R.A. (2009). The basics of epithelial–mesenchymal transition. J. Clin. Investig..

[21] Zavadil J., Böttinger E.P. (2005). TGF-β and epithelial-to-mesenchymal transitions. Oncogene.

[22] Serhan C.N. (2014). Pro-resolving lipid mediators are leads for resolution physiology. Nature.

[23] Headland S.E., Norling L.V. (2015). The resolution of inflammation: Principles and challenges. Semin. Immunol..

[24] Panigrahy D., Gilligan M.M., Serhan C.N., Kashfi K. (2021). Resolution of inflammation: An organizing principle in biology and medicine. Pharmacol. Ther..

[25] Godson C., Guiry P.J., Brennan E.P. (2023). Lipoxin mimetics and the resolution of inflammation. Annu. Rev. Pharmacol. Toxicol..

[26] Chandrasekharan J.A., Sharma-Walia N. (2015). Lipoxins: Nature’s way to resolve inflammation. J. Inflamm. Res..

[27] Bäck M., Powell W.S., Dahlén S.E., Drazen J.M., Evans J.F., Serhan C.N., Shimizu T., Yokomizo T., Rovati G.E. (2014). Update on leukotriene, lipoxin and oxoeicosanoid receptors: IUPHAR Review 7. Br. J. Pharmacol..

[28] Jeong Y.S., Bae Y.S. (2020). Formyl peptide receptors in the mucosal immune system. Exp. Mol. Med..

[29] Zhou Y., You C.G. (2022). Lipoxin alleviates oxidative stress: A state-of-the-art review. Inflamm. Res..

[30] Fu T., Mohan M., Brennan E.P., Woodman O.L., Godson C., Kantharidis P., Ritchie R.H., Qin C.X. (2020). Therapeutic potential of lipoxin A_4_ in chronic inflammation: Focus on cardiometabolic disease. ACS Pharmacol. Transl. Sci..

[31] Kim H., Park S.H., Han S.Y., Lee Y.S., Cho J., Kim J.M. (2020). LXA_4_–FPR2 signaling regulates radiation-induced pulmonary fibrosis via crosstalk with TGF-β/Smad signaling. Cell Death Dis..

[32] Byrne L., Guiry P.J. (2024). Advances in the chemistry and biology of specialised pro-resolving mediators (SPMs). Molecules.

[33] de Gaetano M., Tighe C., Gahan K., Zanetti A., Chen J., Newson J., Cacace A., Marai M., Gaffney A., Brennan E. (2021). Asymmetric synthesis and biological screening of quinoxaline-containing synthetic lipoxin A_4_ mimetics (QNX-sLXms). J. Med. Chem..

[34] O’Sullivan T.P., Vallin K.S.A., Ali Shah S.T., Fakhry J., Maderna P., Scannell M., Sampaio A.L., Perretti M., Godson C., Guiry P.J. (2007). Aromatic lipoxin A_4_ and lipoxin B_4_ analogues display potent biological activities. J. Med. Chem..

[35] Sun Y.P., Tjonahen E., Keledjian R., Zhu M., Yang R., Recchiuti A., Pillai P.S., Petasis N.A., Serhan C.N. (2009). Anti-inflammatory and pro-resolving properties of benzo-lipoxin A_4_ analogs. Prostaglandins Leukot. Essent. Fat. Acids.

[36] Andrews D., Godson C. (2021). Lipoxins and synthetic lipoxin mimetics: Therapeutic potential in renal diseases. Biochim. Biophys. Acta Mol. Cell Biol. Lipids.

[37] Brennan E.P., Mohan M., McClelland A., Tikellis C., Ziemann M., Kaspi A., Gray S.P., Pickering R., Tan S.M., Ali-Shah S.T. (2018). Lipoxins regulate the early growth response-1 network and reverse diabetic kidney disease. J. Am. Soc. Nephrol..

[38] Hong Z.Y., Eun S.H., Park K., Choi W.H., Lee J.I., Lee E.J., Lee J.M., Story M.D., Cho J. (2014). Development of a small animal model to simulate clinical stereotactic body radiotherapy-induced central and peripheral lung injuries. J. Radiat. Res..

[39] Min S.H., Shin J.H., Park S., Cui R., Hur Y.J., Jeong W.H., Kim S.Y., Na Y., Cho J. (2025). Modulation of AMPK/NLRP3 signaling mitigates radiation-induced lung inflammation by a synthetic lipoxin A4 analogue. Int. J. Mol. Sci..

[40] Farh M.E.-A., Kim H.J., Kim S.Y., Lee J.H., Lee H., Cui R., Han S., Kim D.W., Park S., Cho J. (2024). Transcriptional changes in radiation-induced lung injury: A comparative analysis of two radiation doses for preclinical research. Int. J. Mol. Sci..

[41] Corminboeuf O., Leroy X. (2015). FPR2/ALXR agonists and the resolution of inflammation. J. Med. Chem..

[42] Serhan C.N., Levy B.D. (2018). Resolvins in inflammation: Emergence of the pro-resolving superfamily of mediators. J. Clin. Investig..

[43] Serhan C.N., Chiang N., Dalli J., Levy B.D. (2015). Lipid mediators in the resolution of inflammation. Cold Spring Harb. Perspect. Biol..

[44] Fullerton J.N., Gilroy D.W. (2016). Resolution of inflammation: A new therapeutic frontier. Nat. Rev. Drug Discov..

[45] Maderna P., Godson C. (2009). Lipoxins: Resolutionary road. Br. J. Pharmacol..

