# Supplementary material for: Early CYNC-1 Administration Attenuates Radiation-Induced Lung Inflammation and Subsequent Fibrotic Remodeling
Source: Pharmaceutics. 2026 Oct 18;18(10):1321. doi: 10.3390/pharmaceutics18101321 (PMC13708184; doi:10.3390/pharmaceutics18101321)
Supplement: Supplementary file 1 [file pharmaceutics-18-01321-s001.zip › Supplementary_S1_NMR_FINAL.pdf]

Supplementary S1
NMR Characterization of CYNC-1

The ^{1}H and ^{13}C NMR spectra of CYNC-1 are presented in Figures S1 and S2, respectively. Acquisition parameters are summarized in Table S1. HPLC methods and storage measurements are presented together in the supplementary document accompanying Table S2.

NMR measurements

Spectra were recorded using a Varian AS 400 spectrometer at Ewha Womans University. The acquisition headers identify the solvent as DMSO (recorded as “dmso”) and the temperature as 25.0 °C. Tetramethylsilane was used as the internal standard according to the experimental description. Chemical shifts (δ) are expressed in ppm; coupling constants (J), when reported, are expressed in Hz.

Table S1. NMR acquisition parameters for CYNC-1

Recorded parameter	^{1}H NMR	^{13}C NMR
Spectrometer frequency	400.035 MHz	100.600 MHz
Temperature	25.0 °C	25.0 °C
Solvent entry	dmso	dmso
Acquisition date	15 March 2024	15 March 2024
Number of transients (nt)	16	1000
Acquisition time (at)	4.000 s	1.000 s
Relaxation delay (d1)	1.000 s	2.000 s
Spectral width (sw)	4807.7 Hz	25000.0 Hz

The full ^{1}H and ^{13}C spectra include the acquisition headers, chemical-shift axes, and the peak-picking and integration labels displayed in the original records.

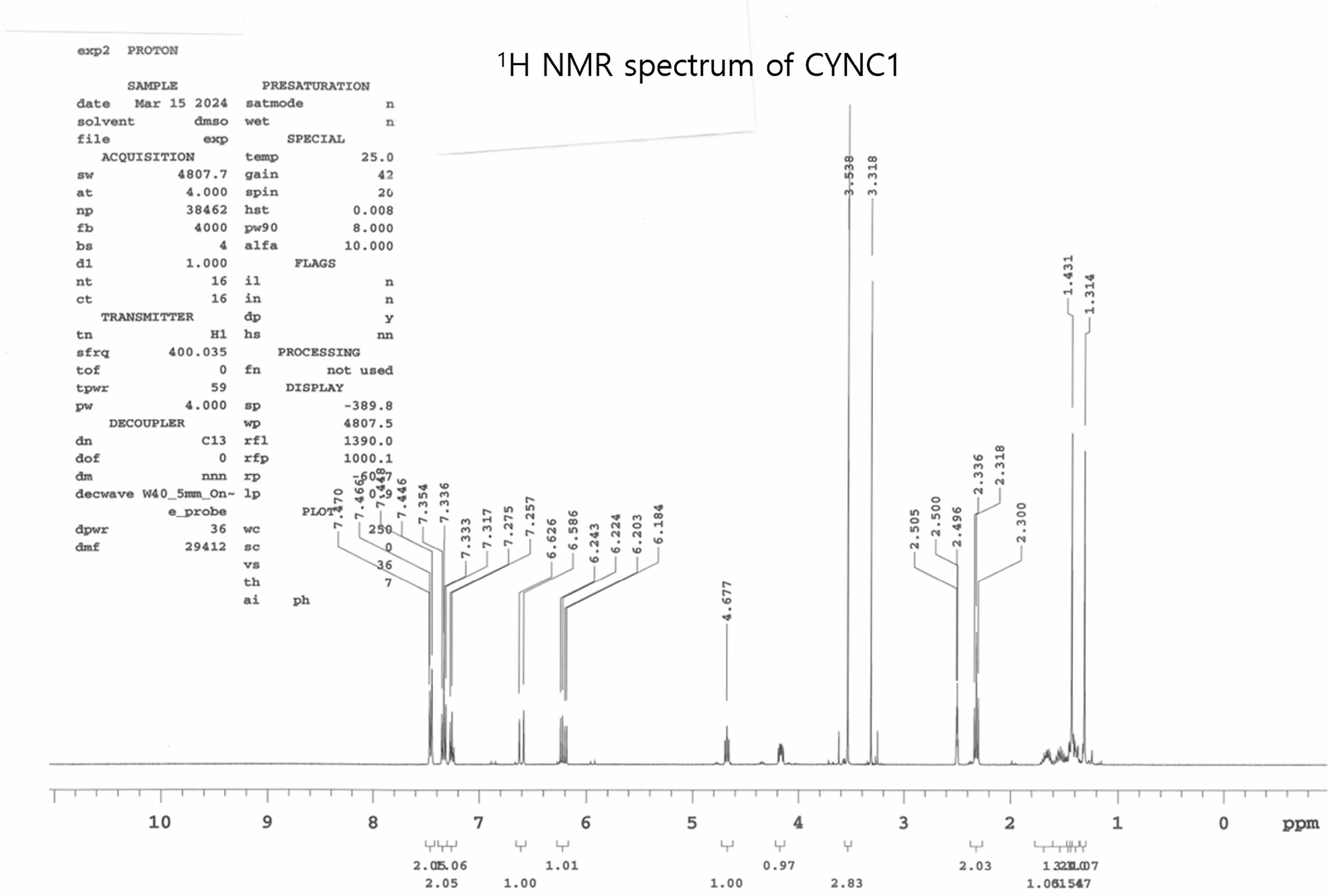

Figure S1. ¹H NMR spectrum of CYNC-1 recorded at 400.035 MHz in DMSO at 25.0 °C.

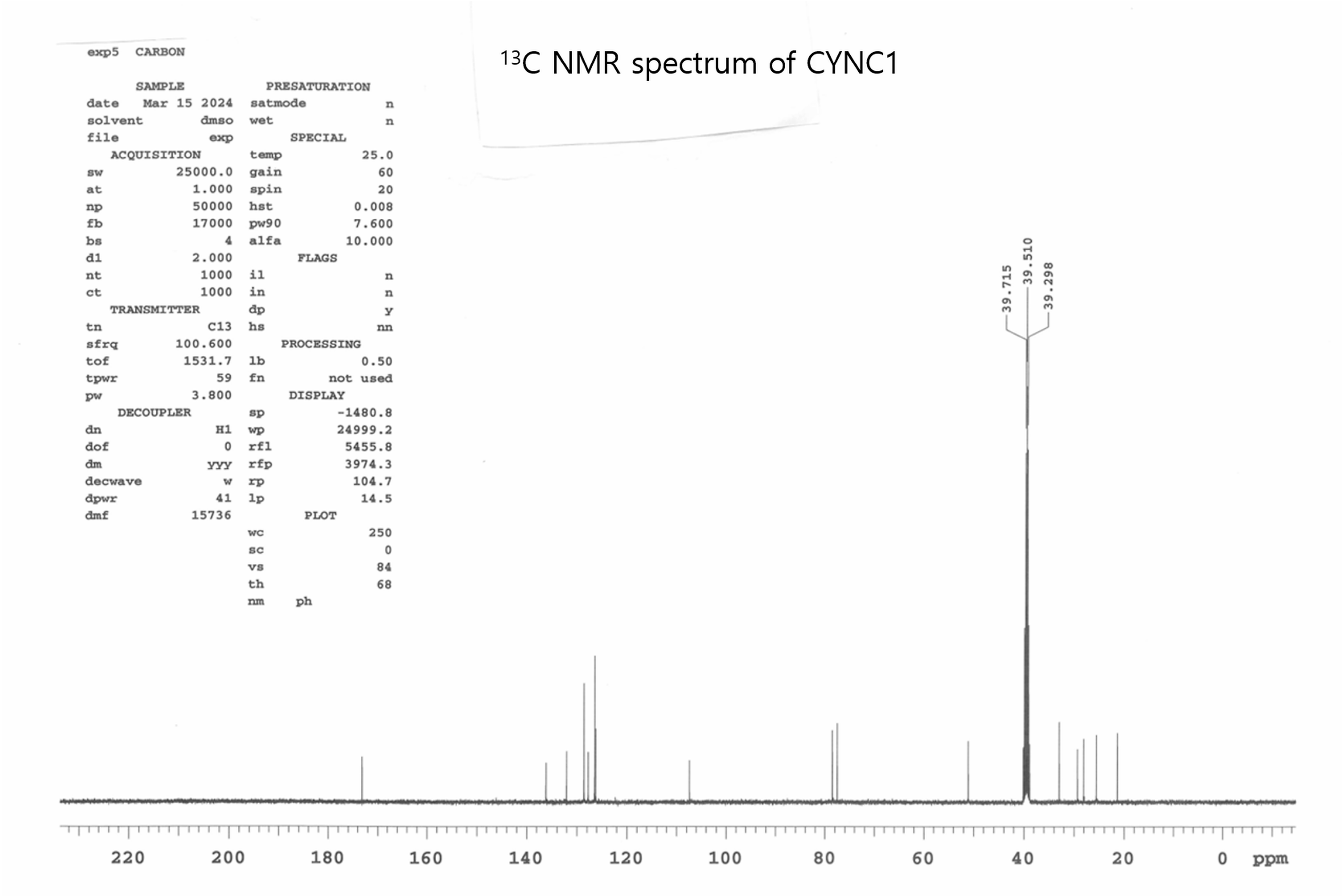

Figure S2. ^{13}C NMR spectrum of CYNC-1 recorded at 100.600 MHz in DMSO at 25.0 °C.
