# Supplementary material for: Early CYNC-1 Administration Attenuates Radiation-Induced Lung Inflammation and Subsequent Fibrotic Remodeling
Source: Pharmaceutics. 2026 Oct 18;18(10):1321. doi: 10.3390/pharmaceutics18101321 (PMC13708184; doi:10.3390/pharmaceutics18101321)
Supplement: Supplementary file 1 [file pharmaceutics-18-01321-s001.zip › Supplementary_S2_HPLC_FINAL.pdf]

# Supplementary S2
# HPLC Purity and Storage Assessment

Table S2 presents the analytical HPLC conditions (panel A) and ethanol-storage measurements over 48 h at −78, −20, and 4 °C (panel B). Preparation and sampling conditions are described below. The reported values are chromatographic main-peak area percentages at 254 nm.

## HPLC analysis and calculation of purity

A Shimadzu LC-2050C 3D HPLC system with a Shimadzu SPD-M20A diode-array detector and a SunFire C18 column (4.6 × 150 mm, 5 µm) was used. HPLC-grade water and acetonitrile were used without further distillation; no buffer was added. The CYNC-1 product-purity analysis used water/acetonitrile (30:70, v/v), a flow rate of 1.0 mL/min, and a 15-min run. The synthesis-related retention time and purity were 5.13 min and 99.7%, respectively.

Although native LXA4 (nLXA4; laboratory designation nLX) has an absorption maximum near 300 nm, impurities were observed at 254 nm. Both nLXA4 and CYNC-1 (laboratory designation LXA1) were therefore evaluated at 254 nm. Chromatographic purity (Area %) was calculated as 100 × analyte peak area / sum of integrated peak areas in the 254-nm chromatogram. This area fraction was not normalized to the initial amount of compound.

### Table S2. HPLC analytical conditions and ethanol-storage assessment

### S2A. Analytical conditions

| Parameter | Native LXA4 | CYNC-1 |
|---|---|---|
| Mobile phase A / B | Water / acetonitrile | Water / acetonitrile |
| Isocratic A:B (v/v) | 40:60 | 30:70 |
| Purity-evaluation wavelength | 254 nm | 254 nm |
| Flow rate | 1.0 mL/min | 1.0 mL/min |
| Run time | 15 min | 15 min |
| Injection volume and mode | 10 µL, manual | 10 µL, manual |
| Sample solvent | Ethanol | Ethanol |
| Buffer / light protection | No buffer / light-protective containers | No buffer / light-protective containers |

## Preparation and sampling

nLXA4 was prepared at 142 µM (0.005%, w/v; 50 µg/mL), and CYNC-1 at 164 µM (0.005%, w/v; 50 µg/mL). Samples were stored in ethanol in light-protective containers at −78, −20, or 4 °C, without buffer or controlled aqueous pH. Two vials were assigned to each compound at each temperature. Vial 1 was sampled consecutively at 3, 6, and 9 h; Vial 2 was sampled at 24 and 48 h. Samples were brought to room temperature after retrieval and promptly injected manually (10 µL), once at each sampling time. The baseline values and all reported storage measurements appear in panel B.

### S2B. Main-peak area percentages at 254 nm over 48 h

| Compound | Storage temperature (°C) | 0 h | 3 h | 6 h | 9 h | 24 h | 48 h |
|---|---|---|---|---|---|---|---|
| nLXA4 (254 nm) | −78 | 91.379 | 91.849 | 92.545 | 89.192 | 89.112 | 88.377 |
| nLXA4 (254 nm) | −20 | 91.379 | 89.865 | 90.306 | 90.061 | 90.550 | 90.671 |
| nLXA4 (254 nm) | 4 | 91.379 | 91.169 | 83.338 | 81.559 | 87.395 | 87.097 |
| CYNC-1 (254 nm) | −78 | 99.595 | 100.00 | 99.818 | 99.686 | 99.542 | 99.542 |
| CYNC-1 (254 nm) | −20 | 99.595 | 100.00 | 99.840 | 99.934 | 99.771 | 99.726 |
| CYNC-1 (254 nm) | 4 | 99.595 | 99.482 | 99.318 | 99.370 | 98.779 | 98.441 |

Values are chromatographic Area %, measured during ethanol storage under light protection. Each post-baseline value represents a single measurement. No SD, SEM, or inferential comparison is reported for this time course.

Test concentrations: nLXA4, 142 µM (0.005%, w/v); CYNC-1, 164 µM (0.005%, w/v). The same baseline value is repeated across the three temperature rows for each compound; these entries do not establish three independent baseline measurements. Measurements at 3, 6, and 9 h share one vial; measurements at 24 and 48 h share another vial.

### Interpretation

CYNC-1 retained high chromatographic area purity during ethanol storage at −78, −20, and 4 °C over 48 h under light-protected conditions. At baseline, the main-peak area percentages were 99.595% for CYNC-1 and 91.379% for nLXA4. After 48 h at 4 °C, the corresponding values were 98.441% and 87.097%; at −20 °C, they were 99.726% and 90.671%; and at −78 °C, they were 99.542% and 88.377%, respectively. Different starting concentrations and purities, compound-specific mobile phases and UV responses, repeated vial sampling, and non-monotonic values limit quantitative comparison of degradation between compounds. These single measurements are descriptive and do not establish independent storage replication. Area % does not quantify absolute recovery or a degradation half-life. Storage in light-protective containers in unbuffered ethanol does not establish photostability, controlled-pH stability, metabolic stability, or systemic exposure.
