# Supplementary material for: Early CYNC-1 Administration Attenuates Radiation-Induced Lung Inflammation and Subsequent Fibrotic Remodeling
Source: Pharmaceutics. 2026 Oct 18;18(10):1321. doi: 10.3390/pharmaceutics18101321 (PMC13708184; doi:10.3390/pharmaceutics18101321)
Supplement: Supplementary file 1 [file pharmaceutics-18-01321-s001.zip › Supplementary_S3_Primers_FINAL.pdf]

# Supplementary S3
# Target Gene Primers for RT-qPCR

Primer sequences for the target genes measured by RT-qPCR in L132 cells. All sequences are written in the 5′ to 3′ direction.

**Table S3. Target gene primers for RT-qPCR**

| Gene | Primer | Sequence (5′ to 3′) |
|---|---|---|
| *TNF* | F | 5′-GGAGAAGGGTGACCGACT-3′ |
| *TNF* | R | 5′-CTGCCCAGACTCGGCAA-3′ |
| *IL1B* | F | 5′-TACCTGTCCTGCGTGTTGAAA-3′ |
| *IL1B* | R | 5′-CTGCTTGAGAGGTGCTGATGT-3′ |
| *IL6* | F | 5′-GTCAGGGGTGGTTATTGC-3′ |
| *IL6* | R | 5′-AGTGAGGAACAAGCCAGA-3′ |
| *CXCL8* | F | 5′-CTTGTCATTGCCAGCTGTGT-3′ |
| *CXCL8* | R | 5′-TGACTGTGGAGTTTTGGCTG-3′ |
| *TGFB1* | F | 5′-GCAGCACGTGGAGCTGTA-3′ |
| *TGFB1* | R | 5′-CAGCCGGTTGCTGAGGTA-3′ |
| *IL10* | F | 5′-GCACTACCAAAGCCACAAAGCAG-3′ |
| *IL10* | R | 5′-GTCAGTAAAGCAGGCAGCATAGC-3′ |

F, forward primer; R, reverse primer. RT-qPCR, quantitative reverse-transcription polymerase chain reaction.
