# Supplementary material for: Early CYNC-1 Administration Attenuates Radiation-Induced Lung Inflammation and Subsequent Fibrotic Remodeling
Source: Pharmaceutics. 2026 Oct 18;18(10):1321. doi: 10.3390/pharmaceutics18101321 (PMC13708184; doi:10.3390/pharmaceutics18101321)
Supplement: Supplementary file 1 [file pharmaceutics-18-01321-s001.zip › Supplementary_S4_Dose-evaluation_FINAL_FINAL.pdf]

Supplementary S4

Preliminary dose-selection study of CYNC-1 after lung irradiation

An independent preliminary dose-selection experiment was conducted before the main experiments. Mice received CYNC-1 at 0.5, 2.5, or 5 mg/kg for two weeks beginning immediately after irradiation. Figure S3 presents the exploratory dose-ranging micro-CT images and damaged-region-volume analysis. Tables S4A–S4C provide group summaries, individual measurements, and exploratory statistical comparisons from this experiment (24 mice across six groups; n = 4 per group).

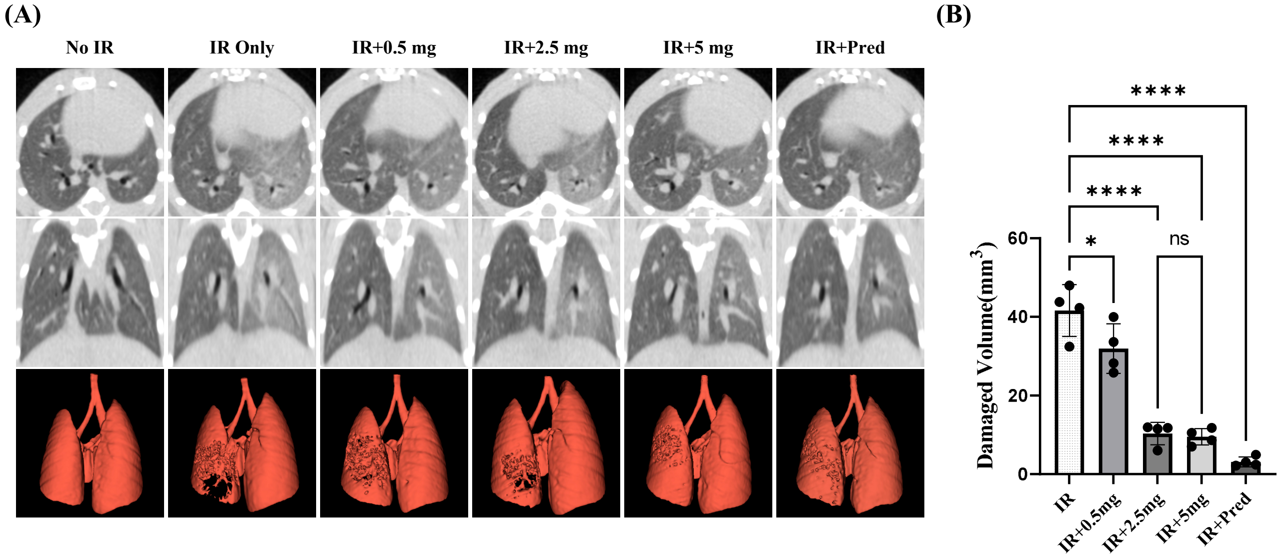

Figure S3. Exploratory dose-ranging evaluation of CYNC-1 after lung irradiation (A) Representative micro-CT images obtained two weeks after irradiation. Axial, coronal, and three-dimensional reconstructed lung images are shown for each treatment group: No IR, IR, IR + CYNC-1 (0.5, 2.5, or 5 mg/kg), and IR + prednisolone (Pred; 2 mg/kg).
(B) Quantification of damaged-region volume measured by micro-CT. Each dot represents an individual mouse, and bars indicate mean ± SD (n = 4 per group). Ordinary one-way ANOVA included all five irradiated groups (20 mice), followed by Tukey's adjustment across all ten pairwise comparisons; selected comparisons are displayed. *P < 0.05, **P < 0.01, ***P < 0.001, ****P < 0.0001; ns, adjusted P ≥ 0.05. The non-irradiated control group was excluded from this analysis because its damaged-region volumes were not reported. Adjusted P values and the unequal-variance sensitivity analysis are provided in Table S4C.

Table S4A. Group summary

Group	n	Total volume (mm^3)	Damaged-region volume (mm^3)	Damaged-region ratio (%)
No IR	4	416.04 ± 23.08	-	-
IR	4	387.38 ± 24.24	41.62 ± 6.58	10.80 ± 1.99
IR + CYNC-1 0.5 mg/kg	4	406.38 ± 17.69	31.90 ± 6.30	7.85 ± 1.54
IR + CYNC-1 2.5 mg/kg	4	435.25 ± 32.18	10.31 ± 2.85	2.38 ± 0.71
IR + CYNC-1 5 mg/kg	4	386.22 ± 56.66	9.50 ± 2.09	2.55 ± 0.90
IR + Pred 2 mg/kg	4	380.65 ± 51.68	3.12 ± 1.24	0.84 ± 0.36

Values are mean ± sample SD. n = 4 for total volume in all groups and for injury-related measurements in the irradiated groups. The damaged-region ratio (%) was calculated as 100 × damaged-region volume/total volume. Pred, prednisolone. No missing values were replaced with zero. "–" indicates that damaged-region volume was not reported and the corresponding ratio was not calculated.

Experimental protocol

Male C57BL/6 mice were obtained at 7 weeks of age and acclimatized for one week. Irradiated groups received a single 75-Gy dose to the left lung through a 5-mm circular collimator, with the remainder of the body shielded. CYNC-1 (0.5, 2.5, or 5 mg/kg, intraperitoneally) or prednisolone (2 mg/kg, orally) was administered every other day for 2 weeks, beginning immediately after irradiation (Day 0). Micro-CT was performed at 2 weeks. Experimental procedures are described in Methods, Sections 2.6–2.7. The numerical records constitute one independent preliminary experiment and are separate from the main-study cohorts.

Table S4B. Individual micro-CT measurements

Group	Animal ID	Vtotal (mm^3)	Vdamage (mm^3)	Damaged-region ratio (%)
No IR	3815	395.705	-	-
No IR	3816	396.803	-	-
No IR	3817	431.798	-	-
No IR	3818	439.846	-	-
IR	3819	389.859	47.983	12.31
IR	3820	396.129	32.438	8.19
IR	3821	353.345	43.785	12.39
IR	3822	410.206	42.289	10.31
CYNC-1 0.5 mg/kg	3856	419.614	28.214	6.72
CYNC-1 0.5 mg/kg	3857	400.686	39.984	9.98
CYNC-1 0.5 mg/kg	3858	421.372	33.618	7.98
CYNC-1 0.5 mg/kg	3859	383.867	25.800	6.72
CYNC-1 2.5 mg/kg	3861	394.320	11.752	2.98
CYNC-1 2.5 mg/kg	3862	433.949	11.926	2.75
CYNC-1 2.5 mg/kg	3863	439.996	6.047	1.37
CYNC-1 2.5 mg/kg	3864	472.754	11.513	2.44
CYNC-1 5 mg/kg	3827	320.382	11.793	3.68
CYNC-1 5 mg/kg	3828	455.110	8.645	1.90
CYNC-1 5 mg/kg	3829	401.192	7.035	1.75
CYNC-1 5 mg/kg	3830	368.195	10.535	2.86
Pred 2 mg/kg	3871	315.293	3.001	0.95
Pred 2 mg/kg	3872	440.574	2.407	0.55
Pred 2 mg/kg	3873	391.588	2.164	0.55
Pred 2 mg/kg	3874	375.136	4.904	1.31

All CYNC-1 and Pred groups received irradiation. Volumes are shown to three decimal places. Individual damaged-region ratios were calculated as 100 × Vdamage/Vtotal and are displayed to two decimal places. Group summaries and statistical tests use the listed component volumes; ratios were calculated before rounding. Animal IDs identify distinct mice and do not imply paired observations across groups. "–" indicates that damaged-region volume was not reported and the corresponding ratio was not calculated.

Table S4C. Statistical comparisons of damaged-region volume

Ordinary one-way ANOVA of damaged-region volume across the five irradiated groups (n = 4 per group; 20 mice): $F(4, 15) = 56.469$, $p = 7.279621 \times 10^{-9}$. Tukey's adjustment covers all ten pairwise comparisons. Given unequal observed SDs, Welch's ANOVA [$F(4, 6.966) = 43.395$, $p = 5.183997 \times 10^{-5}$] with Games–Howell comparisons was also performed as a sensitivity analysis. Calculations used SciPy 1.17.0 in Python.

Comparison	Tukey-adjusted p	Games–Howell adjusted p
IR vs. CYNC-1 0.5 mg/kg	0.047069	0.316016
IR vs. CYNC-1 2.5 mg/kg	4.111708×10^{-7}	0.004119
IR vs. CYNC-1 5 mg/kg	2.941446×10^{-7}	0.005353
IR vs. Pred 2 mg/kg	2.565134×10^{-8}	0.004225
CYNC-1 0.5 mg/kg vs. CYNC-1 2.5 mg/kg	4.087430×10^{-5}	0.013734
CYNC-1 0.5 mg/kg vs. CYNC-1 5 mg/kg	2.669472×10^{-5}	0.015219
CYNC-1 0.5 mg/kg vs. Pred 2 mg/kg	1.225623×10^{-6}	0.008964
CYNC-1 2.5 mg/kg vs. CYNC-1 5 mg/kg	0.998885	0.988544
CYNC-1 2.5 mg/kg vs. Pred 2 mg/kg	0.195752	0.041772
CYNC-1 5 mg/kg vs. Pred 2 mg/kg	0.290913	0.018213

All treatment groups received irradiation. The graph shows selected Tukey comparisons; Games–Howell comparisons do not assume equal variances. CYNC-1 at 2.5 and 5 mg/kg and prednisolone had lower damaged-region volumes than IR in both analyses. The IR-versus-CYNC-1 0.5 mg/kg comparison was significant with Tukey ($p = 0.047069$) but not Games–Howell ($p = 0.316016$). Comparisons of CYNC-1 at 2.5 or 5 mg/kg with prednisolone also differed in significance between methods. The key 0.5-versus-2.5 and 2.5-versus-5 mg/kg dose comparisons were consistent across methods. Results are exploratory and do not establish dose or comparator-drug equivalence or non-inferiority. Total volume and damaged-region ratio are descriptive outcomes without inferential comparisons.

Dose-selection rationale

Damaged-region volume was lower at 2.5 mg/kg than at 0.5 mg/kg (10.31 ± 2.85 versus 31.90 ± 6.30 mm³; mean ± sample SD; Tukey-adjusted $p = 4.087430 \times 10^{-5}$), indicating a greater observed injury-attenuating effect. Increasing the dose to 5 mg/kg produced little additional numerical improvement (9.50 ± 2.09 mm³; Tukey-adjusted $p = 0.998885$ versus 2.5 mg/kg). This pattern suggested an apparent plateau of the observed response over 2.5–5 mg/kg and supported selection of 2.5 mg/kg as the higher dose for the main experiments, with 0.5 mg/kg retained as a lower-dose comparison. The two dose-selection comparisons were consistent in the unequal-variance sensitivity analysis (Games–Howell-adjusted $p = 0.013734$ and 0.988544, respectively).

This small exploratory experiment does not establish dose equivalence, pharmacological saturation, an optimal dose, or tolerability. A non-significant difference between 2.5 and 5 mg/kg is not evidence of equivalence. Treatment began on Day 0, so these data address attenuation of early radiation injury.
